# Fabrication of Sub-50 nm Three-Dimensional Rhombic Zero-Depth PDMS Nanopores with Enhanced Conductance via Silicon Micro-Blade Molding

**DOI:** 10.3390/mi16121375

**Published:** 2025-12-02

**Authors:** Mohammad Matin Behzadi, Philippe Renaud, Mojtaba Taghipoor

**Affiliations:** 1 Micro Nano Systems Laboratory (MNSL), Department of Mechanical Engineering, Sharif University of Technology, Tehran 1458889694, Iran; mohammadmatin.behzadi@mech.sharif.edu; 2 Microsystem Laboratory 4, École Polytechnique Fédérale de Lausanne (EPFL), 1015 Lausanne, Switzerland; philippe.renaud@epfl.ch

**Keywords:** resistive pulse sensors, 3D rhombic zero-depth nanopores, silicon micro-blades, nano-positioning system, photolithography, wet etching, finite element modeling

## Abstract

Zero-depth nanopores present a promising solution to the challenges associated with ultrathin membranes used in solid-state resistive pulse sensors for DNA sequencing. Most existing fabrication methods are either complex or lack the nanoscale precision required. In this study, we introduce a cost-effective approach that combines PDMS molding at the intersection of silicon micro-blades with an innovative high-resolution nano-positioning technique. These blades are created through photolithography and a two-step KOH wet etching process, allowing for the formation of sub-50 nm 3D rhombic zero-depth nanopores featuring large vertex angles. To address the limitations of SEM imaging—such as dielectric charging and deformation of PDMS membranes under electron beam exposure—we devised a finite element model (FEM) that correlates electrical conductance with pore size and electrolyte concentration. This model aligns closely with experimental data, yielding a mean absolute percentage error of 3.69%, thereby enabling real-time indirect sizing of the nanopores based on the measured conductance. Additionally, we identified a critical channel length beyond which pore resistance becomes negligible, facilitating a linear relationship between conductance and pore diameter. The nanopores produced using this method exhibited a 2.4-fold increase in conductance compared to earlier designs, highlighting their potential for high-precision DNA sequencing applications.

## 1. Introduction

Over the past decades, resistive pulse sensing (RPS) has emerged as a powerful electrochemical technique for detecting individual particles with high resolution [1,2,3,4,5,6,7,8,9,10,11,12,13,14,15,16,17,18,19]. Micro or nanopores, which connect two electrolyte reservoirs, are key components of every RPS system. They can be classified by their materials as either biological or solid-state [20]. The first breakthrough in biological nanopores was achieved by Kasianowicz in 1996, who successfully employed α-hemolysin to detect individual polynucleotide strands [21]. This milestone sparked wide interest in using RPS for DNA sequencing [22].

Biological nanopores, owing to their nanoscale dimensions and high sensitivity [23,24], have been successfully commercialized for sequencing applications [25]. However, their size is inherently limited, and their reliance on lipid bilayers renders them fragile, leading to poor stability under variations in temperature, voltage, stress, and pH [26]. The superior mechanical, chemical, and thermal robustness of synthetic membranes has driven the development of solid-state nanopores [27,28]. Solid-state pores can be fabricated in a wide variety of shapes, sizes, and materials, and in some cases can be tuned dynamically during experiments [29,30].

Multiple fabrication techniques have been reported, including electrokinetic flow focusing [31], pulling of nanopipettes [32], track etching measurement [33], laser heating [34], penetration of hot gold (Au) particles [35], and micro- and nano-needle imprinting [36,37]. Nonetheless, nanopores alone are inadequate for DNA sequencing. The membrane containing the pore must be extremely thin (referred to as zero-length membranes); otherwise, multiple nucleotides may simultaneously fill the pore and fail to produce distinct signals [38]. To achieve low-aspect-ratio pores, several methods have been employed, such as dry etching [39], laser-assisted photothermal etching [39], ion beam irradiation [40,41], transmission electron microscopy [42], and electric breakdown [43,44]. These nanopores are typically created in pre-thinned membranes [45,46], in ultrathin oxide layers grown by atomic layer deposition (ALD) [47,48,49], or in the two-dimensional (2D) materials with nanometer-scale thickness [50,51,52,53,54,55,56,57,58,59]. Although promising, these methods remain costly, complex, and highly dependent on advanced microfabrication facilities.

In the early 2000s, Fleming [60] and Ling [61] proposed the concept of “zero-depth nanopores,” also known as “slit-pores.” These pores are created at the intersection of two nanoscale channels, effectively eliminating the thickness of a membrane. Luan [62] later showed through numerical simulations that the resistance in slit pores is lower than that in conventional cylindrical pores. Additionally, the conductance scales linearly with the pore width and remains consistent, even when the membrane thickness increases by two orders of magnitude. This finding suggests a promising approach to overcoming the challenges of fabricating ultrathin membranes for DNA sequencing. In 2018, Arjmandi [63] experimentally validated the concept by fabricating “interfacial nanopores” through dissolution of crossing metallic nanorods embedded in polymer slabs, achieving λ-DNA detection with a mathematically zero-thickness structure.

Despite its ingenuity, Arjmandi’s method proved to be complex [63]. To simplify this approach, Ahmadi and Ardebili [64] introduced a purely mechanical technique in 2024, molding polydimethylsiloxane (PDMS) at the intersection of two sharp commercial blades. Their method produced three-dimensional (3D) zero-depth pores ranging from 350 nm to 20 μm in robust, biocompatible PDMS membranes, and successfully detected polystyrene beads and yeast cells. However, limitations arose from the low resolution of their positioning system, and the insufficient sharpness of commercial blades precluded the fabrication of sub-350 nm pores. Additionally, the pores demonstrated lower conductance compared to 2D nanopores, which aligns with numerical studies indicating that a smaller wall angle of the pore reduces ionic transport [65].

In this study, we build upon this foundation by developing a cost-effective and high-precision method for fabricating zero-depth nanopores. We employed microfabricated silicon blades with nanoscale cutting edges and large vertex angles, in combination with a low-noise nano-positioning system, to mold sub-50 nm PDMS pores. Based on both numerical and experimental results, we developed an indirect electrochemical method to estimate pore size. Additionally, we confirmed that an increased blade vertex angle enhances pore conductance. These results represent a key step toward practical, high-performance zero-depth nanopores for next-generation solid-state DNA sequencing.

## 2. Materials and Methods

### 2.1. Micro-Blade Fabrication

Previous work by Ahmadi and Ardebili employed commercial cutter blades (made by OLFA Corporation, Osaka, Japan), which, despite being cost-effective, lack the necessary sharpness for precise nanopore fabrication [64]. Recent numerical simulations have shown that for conical pores, increasing the wall angle leads to an increase in conductance [65]. Since the physics underlying this phenomenon can also be applied to zero-depth pores, we can expect that using blades with a larger vertex angle will allow us to fabricate zero-depth nanopores with higher conductivity.

**Figure 1:**
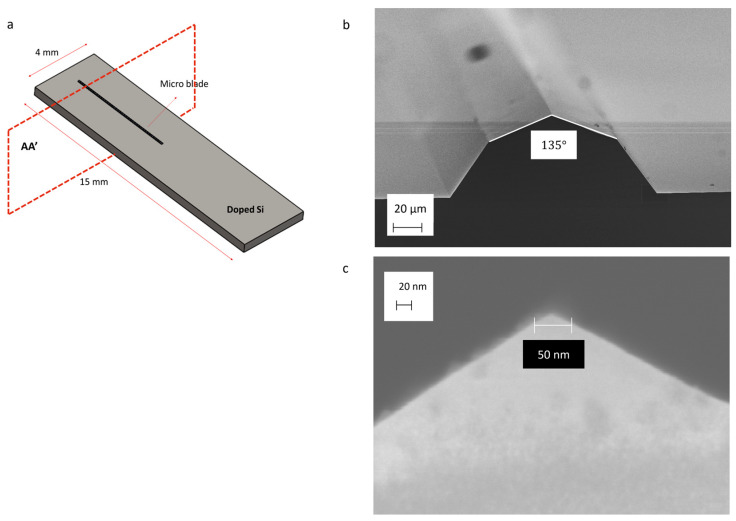
Schematic of the silicon chip and SEM picture of the blade’s section: (**a**) Layout of micro-blade on doped silicon (P-type) chip; (**b**) Section view of the silicon micro-blade (AA’) resulting from the KOH wet etching process. The blade is composed of <111> and <311> crystallographic planes, with a blade tip angle of 135°. (**c**) The diameter of the blade tip is less than 50 nm.

In order to achieve nanopores with a high vertex angle, we utilized silicon micro-blades (Figure 1a) fabricated via photolithography and a two-step wet etching process of <100> P-Boron-doped silicon wafers (100/P/SS/01-05) using potassium hydroxide (KOH). The resistivity of the wafers ranges from 0.1 to 0.5 ohm.cm, and only one side is polished (referred to as single-sided or SS).

Figure 1a illustrates the blade geometry, which has been examined using scanning electron microscopy (SEM). The blade is composed of <111> and <311> crystallographic planes. The tip angle is approximately 135° (Figure 1b), and the diameter of the blade tip is less than 50 nm (Figure 1c). Theoretically, achieving a sub-nanometer tip dimension is also possible in this way. The blades are 6 mm in length and approximately 40 µm in height (Figure S1).

**Figure 2:**
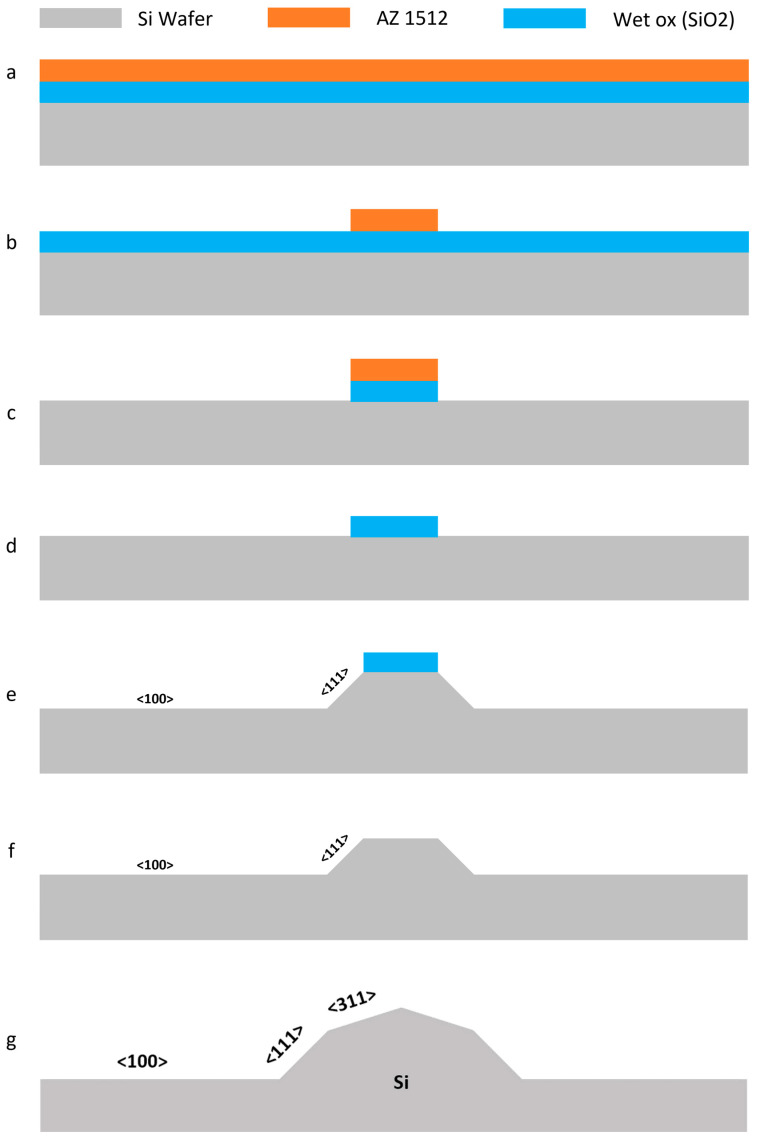
The process flow for fabricating silicon micro-blades using photolithography and a two-step wet etching method with KOH: (**a**) A <100> P-boron-doped silicon wafer is coated with Wet-Ox and AZ 1512 photoresist; (**b**) The sketch of the blades is exposed onto the wafer using a laser writer; (**c**) The Wet-Ox layer undergoes dry etching; (**d**) The photoresist is stripped away using oxygen plasma; (**e**) The first step of silicon wet etching is carried out using KOH. The areas adjacent to the photoresist will be etched along the <111> plane [66]; (**f**) Wet etching of Wet-Ox is performed using HF; (**g**) The second step of silicon wet etching is conducted with KOH, where the free surface of the wafer surrounded by two <111> planes is etched along the <311> planes [67].

The wafers should be coated with 2 µm of Wet-Ox and AZ 1512 Photoresist, respectively. Figure 2 illustrates the process flow for fabricating the micro-blades. A detailed description of the micro-blade fabrication method is provided in the Supplementary Materials (Table S1).

### 2.2. Nano-Positioning Setup

**Figure 3:**
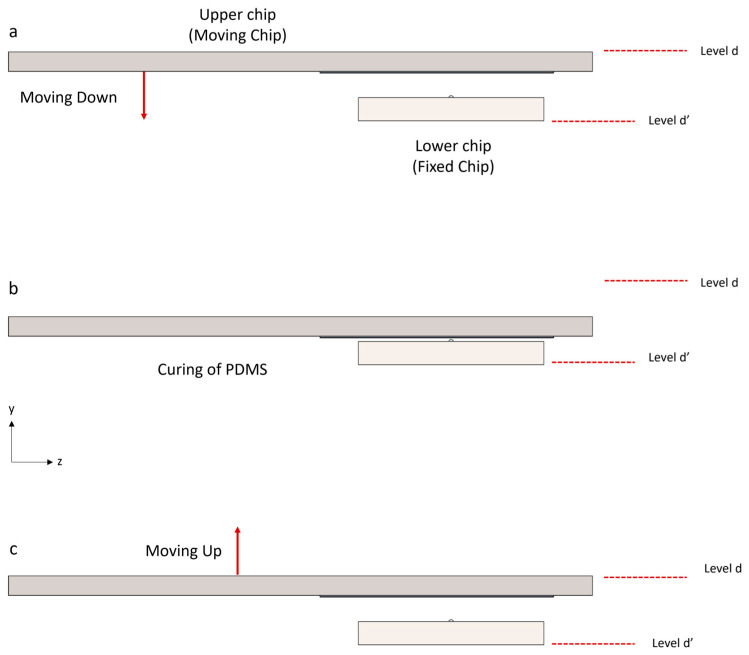
Schematic of the blades’ movement and coincidence. This idea involves molding PDMS at the intersection of two silicon micro-blades. Levels “d” and “d′”, indicated by the red dashed lines, serve as the reference levels for the upper and lower chips, respectively. (**a**) Two silicon chips, each containing a micro-blade, are used. The lower chip is fixed in place, while the upper chip should be smoothly lowered. (**b**) Once the blades collide, the molding process with PDMS begins. During the curing time, the blades remain in tangential contact. (**c**) After the PDMS has been cured, the chips should be carefully separated to detach the membrane (PDMS is not depicted in this figure; the purpose of this figure is just to show the silicon chips’ movement).

To fabricate zero-depth nanopores by molding PDMS at the intersection of blades [64], the following steps must be performed. First, the micro-blades must be positioned relative to each other as skew lines. Then, they should be brought into smooth tangential contact without cutting, breaking, or penetrating one another for the fabrication of the PDMS zero-depth nanopore (Figure 3a). Once the micro-blades collide, molding at the intersection with the PDMS can begin (Figure 3b). PDMS is biocompatible and is impermeable to fluids, making it an excellent material for solid-state nanopore fabrication [68,69,70]. The PDMS must be thoroughly mixed with its curing agent in a 10:1 ratio. Additionally, degassing the PDMS using a desiccator is essential both before and after pouring it onto the chip. After the PDMS has been cured, the chips must be carefully separated to detach the membrane (Figure 3c).

**Figure 4:**
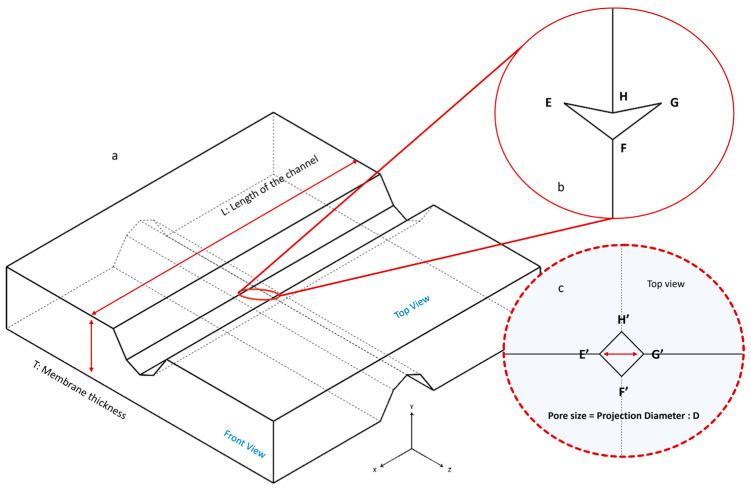
Schematic of the PDMS membrane: (**a**) After separating the chips, the detachable PDMS membrane can be removed from the lower chip. Two crossed channels are created by silicon micro-blades; (**b**) A 3D RZD pore (EFGH) is formed where these two crossed channels intersect; (**c**) When viewed from above, the pore appears as a square (E′F′G′H′). The diameter of this square (E′G′ = F′H′), represents the pore size or pore diameter (D) in this paper.

The PDMS membrane features two crossed channels created using micro-blades (Figure 4). At the intersection of these blades, a 3D rhombic zero-depth (RZD) pore (Figure S4) is formed (EFGH), which projects as a square on the top plane (E′F′G′H′). The diagonal length of the square (E′G′ = F′H′), denoted as “D”, represents the pore size or pore diameter in this paper.

**Figure 5:**
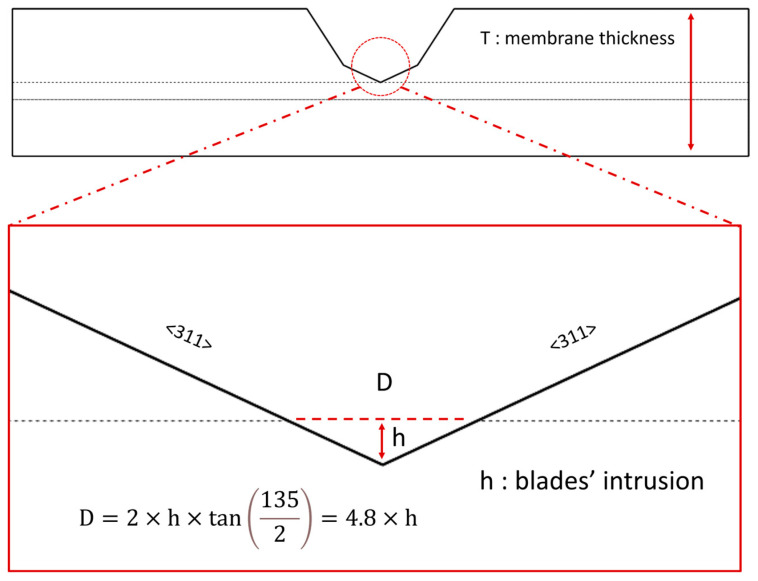
The calculation of pore diameter (D), which depends significantly on the intrusion of the blades (h). The D-h relationship emphasizes the importance of using a high-precision positioning system to ensure that the blades do not cut through one another and stop exactly when they make contact. The silicon blade is composed of <111> and <311> planes. The zoomed image focuses on the <311>-<311> planes.

The pore diameter is highly dependent on the depth to which the blades penetrate one another. When examining the membrane from a frontal view (Figure 5), we can easily calculate the pore diameter as a function of the blades’ intrusion (h) by utilizing trigonometric relationships. It is important to note that the vertex angle of the channel is 135°.

The pore diameter is almost five times larger than the blades’ intrusion depth. The smallest pore size is achieved when the blades are nearly touching each other at their tangents. This highlights the necessity of employing a high-precision positioning system to ensure that the blades do not cut into each other and accurately stop upon making contact. One of the main reasons Ahmadi and Ardebili were unable to achieve nano-scale pores was their reliance on a low-resolution micro-positioning system [64].

**Figure 6:**
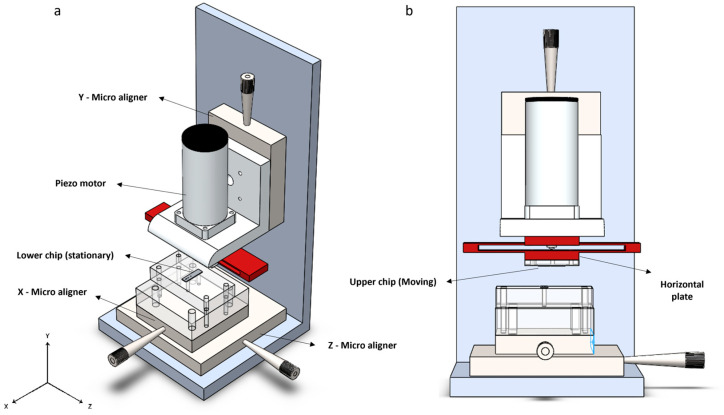
Schematic of the micro-positioning setup: (**a**) An isometric view of the setup. The X and Z micro aligners allow for adjusting the position of the blades relative to each other. The Y micro aligner is used for manual coarse movement. When the chips are sufficiently close, the piezo motor performs fine movements until the blades touch; (**b**) A front view of the setup. The upper chip is mounted on a PMMA plate, which is attached to the horizontal plate of the bending component (shown in red). The motor shaft moves linearly, applying force to the horizontal plate and causing the upper chip to move downward along the Y-axis with high accuracy.

In this study, we employed a novel nano-positioning system that could accurately move the chips. In the design we developed (Figure 6), the “Lower chip” is positioned on a PMMA plate and can move along the X and Z axes using a two-axis PI micro aligner with a resolution of 10 µm. The “Upper chip,” on the other hand, is capable of movement only along the Y axis, with its coarse movement facilitated by a single-axis PI micro aligner, manually, with a resolution of 10 µm too. Fine movement of the upper chip is accomplished using the linear piezo motor LTC 40, which can move 5 µm per full step. With the PMD 301 driver, each full step can be subdivided into 8192 micro-steps, resulting in a nominal resolution of approximately 0.6 nm per micro-step. Although commercial software was provided with the motor, we developed new control code in Python (Version 3.10.4) to enable closed-loop control of the motor movement.

**Figure 7:**
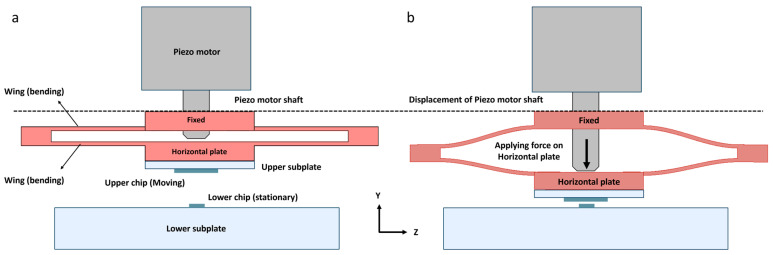
Schematic of the bending component: (**a**) This figure illustrates the bending component, which consists of a fixed part, wings, and a horizontal plate that moves along the *Y*-axis; (**b**) The horizontal plate is shaped like a line tangent to the apex of a parabola. As a result, even though it moves downward, it does not bend.

The upper chip is attached to the “Bending component”, highlighted in red in Figure 6. This component features a “Horizontal Plate” to which the chip is attached, and it is driven by the motor shaft (Figure 7a). Additionally, two thin, elongated wings bend in a parabolic shape. The horizontal plate is designed to be tangent to the apex of the parabola. These wings ensure that the horizontal plate remains horizontal and only moves along the *Y*-axis without bending (Figure 7b). The elastic properties of the wings naturally allow them to return to their original position. The fabrication specifications for the bending component are provided in the Supplementary Materials (Figure S5). The horizontal plate is attached to the shaft of the piezo motor, ensuring that its movement is perfectly synchronized with the shaft. This innovative design, featuring a bending component, helps us avoid issues such as backlash, which is commonly encountered in conventional positioning setups [71,72].

After positioning and aligning the lower chip along the X- and Z-axes, the upper chip is initially lowered manually using the aligner (Figure S2). Once the chips are visually close (Figure S6), the piezo motor is activated to perform fine movement. The motor is programmed to move 6 nm per step (10 micro-steps). When the blades make contact, the molding process begins. It is essential to note that PDMS is poured onto the lower chip before the chips come into contact with each other. After the PDMS has cured, the chips should be carefully separated.

The lower micro aligners’ ability to move along the X- and Z-axes allows us to reuse the chips multiple times. By adjusting the position of the chips along these two axes for each experiment, we can change the collision point of the blades.

### 2.3. Electronic Measurement

**Figure 8:**
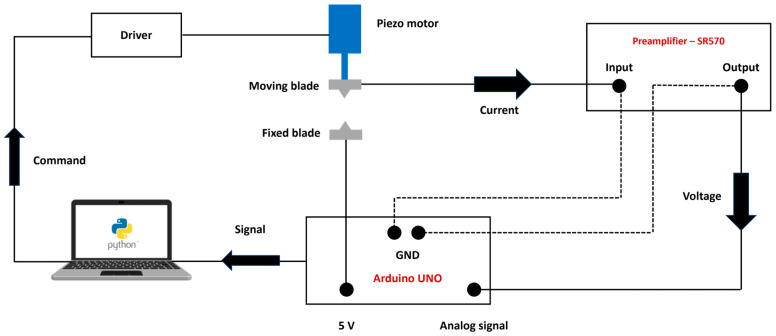
The schematic of the electrical setup is designed to detect the exact moment when the blades make contact. When the blades touch, an electrical current flows through the circuit. The low-noise SR 570 preamplifier measures this current and sends a corresponding voltage signal to the Arduino based on the measured current. The Arduino then relays this signal to the computer, which decides whether to send a stop command to the motor. The preamplifier can measure currents as low as 1pA. By using coaxial cables, BNC connectors, grounding the entire system, and enclosing the entire setup in a Faraday cage, we have reduced the average electrical noise to a maximum of 20 pA.

To create a nanopore, it is essential to detect the precise moment when the blades collide. In this study, we employ an electrical feedback system (Figure 8). An Arduino UNO provides a direct current (DC) voltage of 5 volts. The SR 570 preamplifier measures the minimum current of 100 pA that occurs when the blades come into contact. The preamplifier generates a voltage signal based on the measured current, which is read by one of the Arduino’s analog input ports. The Arduino then forwards this signal to the Python script that controls the movement of the piezo motor. Upon receiving a stop signal from the Arduino, the computer instructs the motor to halt. To minimize electrical noise and ensure accurate current detection, the entire system should be shielded and grounded. The setup should also be housed within a Faraday cage. Coaxial cables and BNC connectors are used in conjunction with the preamplifier.

Vibrations can cause the blades to touch before actual contact is intended, resulting in false stop signals. Additionally, if the blades do come into contact, vibrations may cause them to slide against one another, potentially damaging them and preventing the successful formation of a nanopore (Figure S10). To address this issue, the entire setup should be placed on an active anti-vibration table (Figure S11), and experiments should be conducted in an environment with minimal noise and vibrations.

### 2.4. PDMS Curing

According to the technical documents for Sylgard 184, PDMS typically cures at room temperature within 48 h or at 80 °C in just 4 h. However, based on our experience, considering the small amount of PDMS used in each experiment (100 mg) and the membrane thickness, which is approximately 80 µm (Figure S3), we have found that it can cure at room temperature in just 12 h and only 15 min at 80 °C.

Temperature fluctuations can cause expansion and contraction in various components of the setup, such as chips and blades. Therefore, it is essential to cure PDMS in an environment with a stable and constant temperature. To maintain this stability, we utilize a thermostat equipped with a PID controller manufactured by CENGAGE (Boston, MA, USA). Two 300 W U-shaped heaters are connected in series to the thermostat through a single-phase SSR-25-DA relay. The temperature was measured using a type K-01 thermocouple. The entire system is housed in an aluminum box, which is grounded and also serves as a Faraday cage. With this setup, we can control and limit temperature variation to a maximum of 0.1 °C. We set the temperature to 35 °C and keep the system inside the box for 12 h (Figure S9).

### 2.5. PDMS Adhesion to Silicon

**Figure 9:**
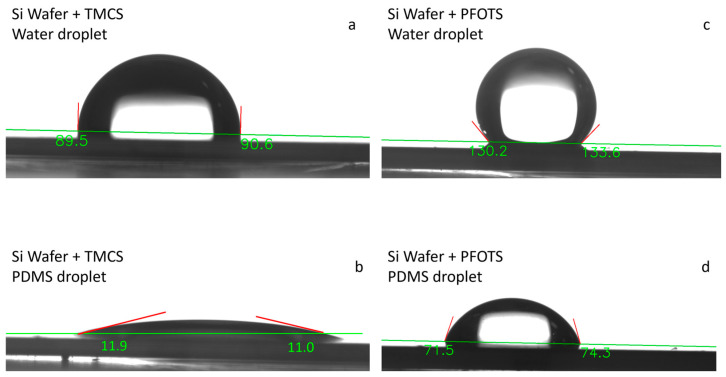
Contact angle of water and PDMS with a silicon wafer with and without silane coating. Additionally, the contact angle of bare silicon with water is measured to be 77° [73]: (**a**) The contact angle of water on a silicon wafer coated with TMCS (Trimethylchlorosilane); (**b**) The contact angle of water on a silicon wafer coated with PFOTS; (**c**) The contact angle of PDMS on a silicon wafer coated with TMCS; (**d**) The contact angle of PDMS on a silicon wafer coated with PFOTS.

Removing and peeling the PDMS membrane from the lower chip after separating the chips can be challenging. PDMS tends to adhere strongly to silicon after curing. To minimize this adhesion, we initially applied Trichloro-1H,1H,2H,2H-perfluorooctyl silane (PFOTS), which is available from Sigma. We placed the silicon chips along with a few droplets of PFOTS in a desiccator for 4 h. Once a thin layer of PFOTS covered the silicon surface, it became highly hydrophobic (Figure 9c), preventing PDMS from sticking to it after curing (Figure 9d).

**Figure 10:**
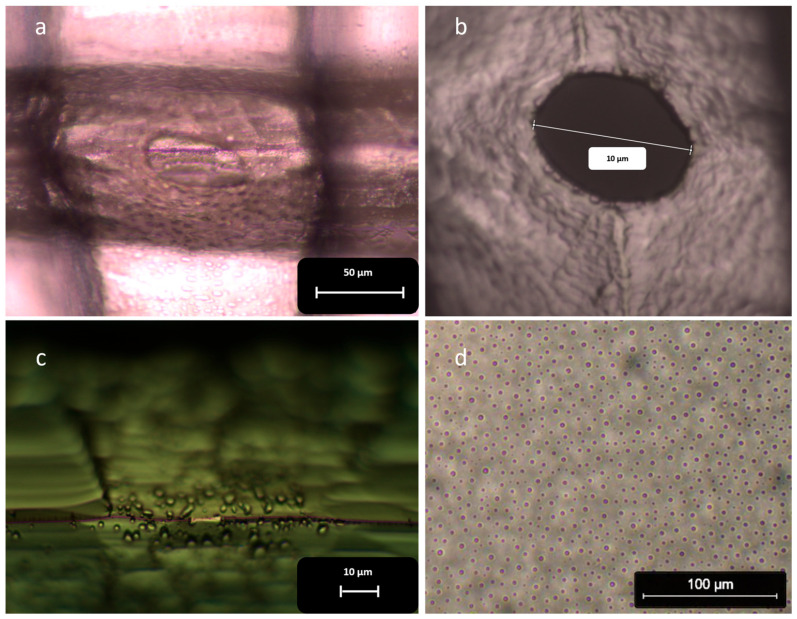
The surface of the chips after using PFOTS and its effect on the shape and size of the pores: (**a**,**b**) The images of the PDMS membrane after separating the chips show unusually large circular or elliptical pores. The shape of these pores suggests they did not form due to the intrusion of the blade; (**c**) PFOTS droplets coat the surface of the blade. The aggregation of these droplets at the collision point prevents PDMS from being present in that area, which inhibits the formation of nanopores; (**d**) Droplets of PFOTS cover the entire surface of the chip.

In all instances where we used PFOTS, we observed unexpectedly large circular or elliptical pores after concluding the process (Figure 10a,b).

**Figure 11:**
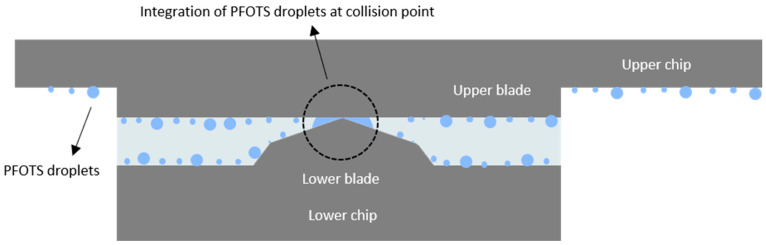
The aggregation of PFOTS droplets at the collision point of the blades. When the blades collide, small PFOTS droplets near the contact point merge to form a larger droplet. This aggregation of PFOTS droplets prevents PDMS from filling the area, leading to the creation of large circular pores.

This issue arose from the presence of PFOTS droplets, which can be observed under a microscope on the surface of the chip and the silicon blades (Figure 10c,d). When the blades come close to each other, the PFOTS droplets at the point of contact merge (Figure 11). This coalescence prevented PDMS from filling the area around the collision point, leading to the formation of large circular pores (Figure 10a,b).

Trimethylchlorosilane (TMCS) was used as an alternative solution. After placing the silicon chips along with a few droplets of TMCS inside the desiccator for about 30 min, a thin layer of TMCS forms on the surface of the silicon chips. This layer allows for the easy removal of the PDMS membrane after curing. Although TMCS is less effective than PFOTS at reducing the adhesion of PDMS to silicon (Figure 9a,b), its performance was sufficient for the purposes of this study. Additionally, TMCS is more cost-effective than PFOTS.

### 2.6. Removing the Membrane

**Figure 12:**
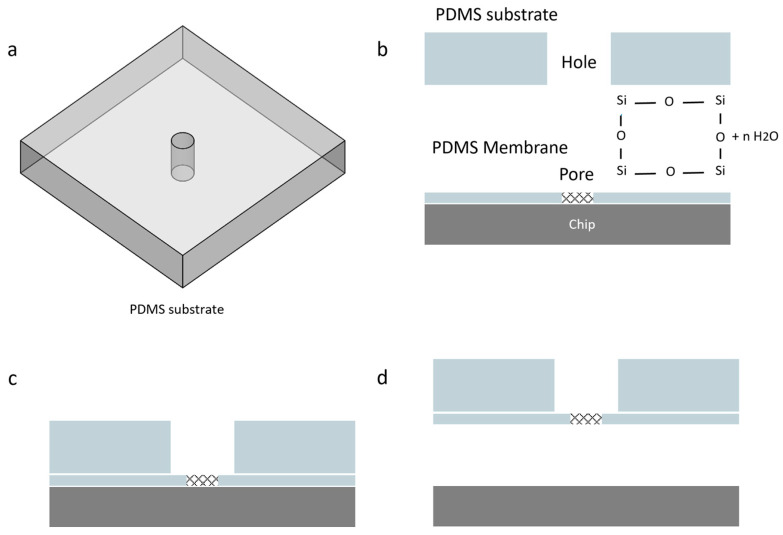
Removing the PDMS membrane using oxygen plasma bonding. This process involves the following steps: (**a**) The PDMS substrate, which has a 3 mm hole at the center, must be prepared; (**b**) The membrane and substrate should be placed into the Diener Femto oxygen plasma for 6 seconds at a pressure of 0.6 mbar to activate the surfaces. It is crucial to ensure that the activation time is neither shorter nor longer; otherwise, the parts may not bond properly. (**c**) After removing the components from the oxygen plasma, the activated surface of the substrate shall be pressed gently onto the membrane. For optimal bonding, it is recommended to place them in an oven at 80 °C for 3 min; (**d**) Finally, by lifting the PDMS substrate, the membrane will detach from the chip.

After curing the PDMS and separating the chips, to remove the membrane from the lower chip, we utilized plasma bonding. A PDMS substrate with a 3 mm hole at its center was prepared. By employing this PDMS substrate along with oxygen plasma bonding, we were able to lift the PDMS membrane while ensuring that the pore remained centered over the hole in the substrate (Figure 12). We used Diener Femto oxygen plasma to activate the surfaces of both the PDMS substrate and the membrane.

## 3. Results and Discussion

### 3.1. Zero-Depth Micro-Nanopores

**Figure 13:**
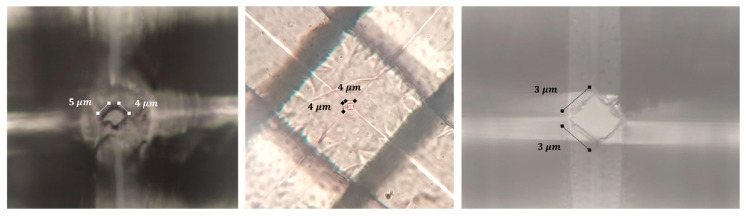
Micro-scale pores observed under the optical microscope appear as squares, which are projections of 3D RZD pores on the top plane. Due to various error-inducing factors, the blades could become damaged or may cut against each other, leading to the formation of larger pores at their intersections.

Several parameters, including low positioning system accuracy, low conductivity of the blades, electrical noise, temperature fluctuations during the curing process, and mechanical vibrations, could lead to the formation of micro-scale pores. Figure 13 illustrates some of the micro-scale pores observed under an optical microscope.

**Figure 14:**
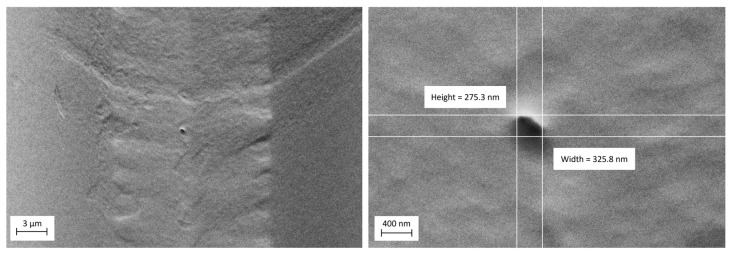
The SEM image shows an RZD nanopore in a PDMS membrane, with a pore size of approximately 300 nm. To enhance image quality, a 5 nm layer of gold was sputtered onto the membrane. This image was taken using a low accelerating voltage (1 kV) with a low probe current (200 pA) to minimize charging and damage. The additional SEM parameters used to acquire this image are provided in the Supplementary Materials (Table S2).

By mitigating these error factors, we successfully fabricated nanopores. As nanopores are not visible with optical microscopes (Figure S13), scanning electron microscopy is necessary for their visualization (Figure 14).

Because PDMS is electrically insulated, it is necessary to sputter a thin layer of gold onto the membrane before SEM imaging. Also, when operating the SEM, it is important to use low-voltage mode; otherwise, the electron charge could damage the membrane and cause variations in pore size. Figure 14 illustrates a nanopore with an approximate size of 300 nm.

Visualizing sub-100 nm 3D RZD structures using SEM is quite challenging. Additionally, once a membrane is imaged, it can no longer be utilized for particle sensing. Therefore, in accordance with the work of Ahmadi and Ardebili [64], we opted to determine nanopore size indirectly via conductance measurements. To this end, we derived a mathematical correlation between pore diameter and conductance using both numerical and experimental methods.

### 3.2. Simulation Result

For a zero-depth pore, the pore resistance is negligible, and the relationship between pore conductance and its diameter, even at the nanoscale range, is linear and independent of membrane thickness [62,63,64]. Ahmadi and Ardebili suggested first measuring the electrical conductance for the micropores that can be visually sized using an optical microscope. By analyzing the results, a linear correlation between pore conductance and pore diameter can be established. Using the derived correlation, the sizes of nanopores can be determined in real time during electrochemical measurements based on their conductance [64].

**Figure 15:**
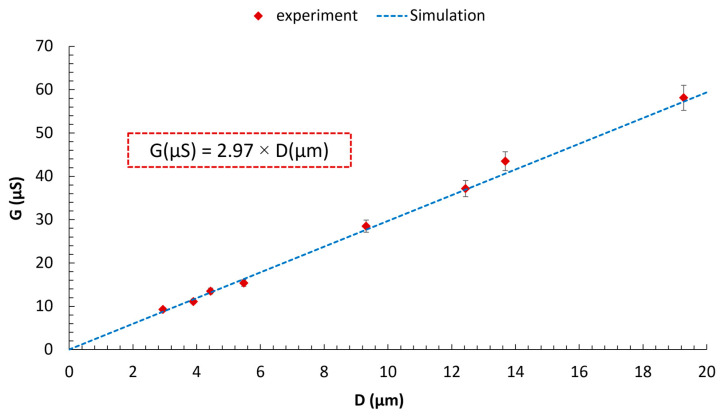
Comparison of numerical and experimental results. In both studies, the concentration of the KCl electrolyte was maintained at 250 mM. For the numerical analysis, a DC voltage of 0.25 V was applied between the electrodes. In contrast, the experimental setup utilized an AC voltage of 0.25 V with a frequency range of 1 to 100 Hz.

Similarly, we measured the conductance of the micro-scale pores, whose sizes were determined through visual sizing using a microscope, with a potentiostat manufactured by Kianshar Co. (Tehran, Iran). During the experimental investigation, an AC voltage of 0.25 V was applied to the Ag/AgCl electrodes, with a variable frequency ranging from 1 to 100 Hz. To minimize the influence of surface charge, simulations and experiments were conducted using a 250 mM KCl solution. As established by Taghipoor et al., at such high electrolyte concentration, the electric double layer (EDL) thickness becomes negligible, and the conductance is dominated by the bulk solution [74]. Furthermore, we numerically investigated the relationship between the pore conductance (G) and its diameter (D). We solved the Poisson, Nernst–Planck, Navier–Stokes, and continuity equations [65,75] simultaneously using the finite element method for a 3D RZD pore, without any particles present. The equations, key parameters, geometry, and more detailed information about the numerical simulation are discussed in the Supplementary Materials (Figures S15 and S16, Tables S4–S6). A mean absolute percentage error (MAPE) of 3.69% between the numerical and experimental results confirms the model’s accuracy (Figure 15). As expected [62,64], a linear relationship is observed between G and D. A key observation is that, under the same conditions, the conductance of the zero-depth pores created using micro-silicon blades is nearly 2.4 times greater than that reported by Ahmadi and Ardebili [64]. This enhancement can be attributed to the larger vertex angle of the channels. As the vertex angle of the channels increases, the folded rhombic pore opens up, causing its geometry to approach that of a 2D pore.

**Figure 16:**
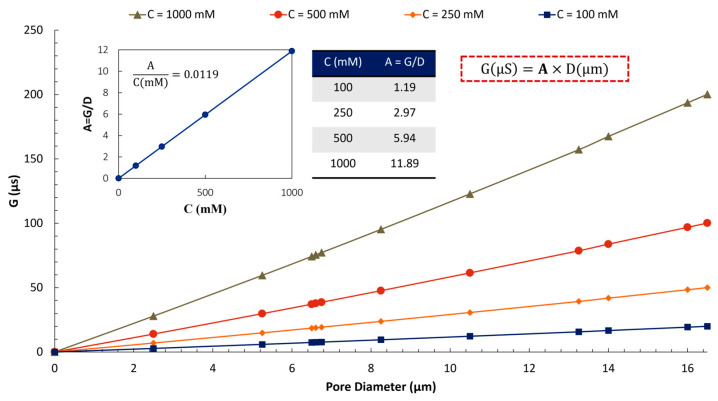
The relationship between pore conductance and diameter at various electrolyte concentrations shows a linear trend. At low concentrations of KCl, the ratio of $\frac{G}{DC}$ remains constant. The simulations were conducted under a quasi-steady state with incompressible electrolyte flow at a DC voltage of 0.25 V [75,78,79,80].

To establish a general correlation (Equation 1), we conducted a numerical investigation of the relationship between G and D at various concentrations (C) of KCl. Figure 16 demonstrates that for low concentrations of KCl, where the conductivity of the solution increases linearly with ionic concentration [76,77], the $\frac{G}{D}$ ratio (A) increases linearly with the electrolyte concentration. The correlation between G and D at any given KCl concentration is linear, too.

Equation (1) is applicable only for channel lengths exceeding a critical value. Our analysis indicates that not all pores formed at channel intersections can be considered “Zero-depth,” as pore resistance and membrane thickness cannot be disregarded for shorter channels.

(1) $$G\left(\mu S\right)=0.0119\times C\left(mM\right)\times D(\mu m)$$

**Figure 17:**
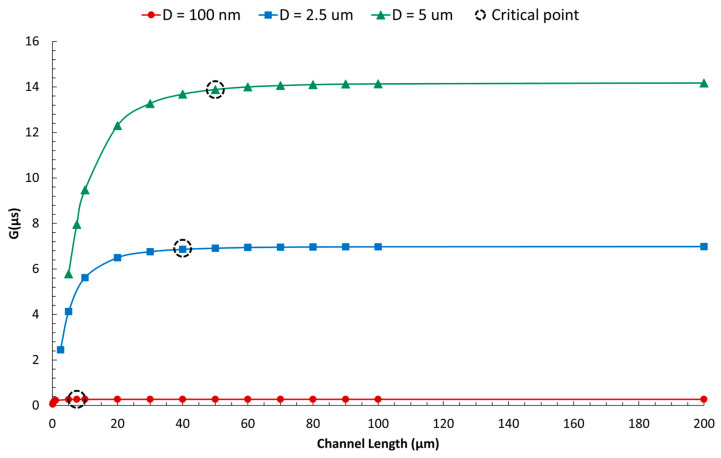
Numerical investigation of the variation in pore conductance with channel length. When the length of the channel exceeds a critical value, pore conductance stabilizes to a constant level, allowing us to disregard pore resistance. The critical length (L_C_) is defined as the channel length beyond which the change in pore conductance is less than 1% (Figure S17). When the channel length is smaller than the critical value, the G-D relation is nonlinear.

Figure 17 illustrates that reducing the channel length below a certain value, termed the “Critical length,” results in a decrease in pore conductance. In other words, pore resistance becomes negligible only when the channel length exceeds the Critical length.

The derived Equation (2) estimates the Critical length (L_C_) for the specific geometry being studied, indicating that the critical length is proportional to the square root of the pore diameter (D).

(2) $$L_{c}\;\left(\mu m\right)=23.8\times \sqrt{D(\mu m)}$$

### 3.3. Nanopore Fabrication

Equation (1) enabled us to indirectly determine the size of the 3D RZD nanopores using electrochemistry. By utilizing a potentiostat, with a 250 mM potassium chloride solution as the electrolyte and applying an AC voltage of 0.25 V to the electrodes, we were able to experimentally size the sub-50 nm nanopores. Before electrochemical measurements, the inherently hydrophobic PDMS membrane must be rendered hydrophilic, which could be achieved through surface activation via oxygen plasma treatment.

**Figure 18:**
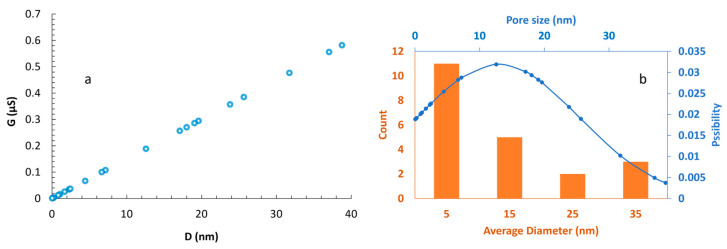
Estimating the size of 3D RZD nanopores based on measured conductance: (**a**) The results from conductance measurements of different tests are presented. The diameter of the nanopores has been estimated using the mathematical correlation of G-D for a concentration of 250 mM KCl, with a 0.25 V AC voltage applied to the Ag/AgCl electrodes; (**b**) This figure illustrates the count and normal distribution of the zero-depth nanopores based on their size. While the sizes of the nanopores vary, most are smaller than 10 nm, with some reaching up to 40 nm.

Following the resolution of initial fabrication challenges, we successfully produced the first sub-50 nm zero-depth nanopore and subsequently conducted 29 additional fabrication trials. From these 30 total tests, 21 resulted in sub-50 nm zero-depth nanopores (Figure 18a), while 9 yielded microscale features (2–5 µm) and were classified as failures, corresponding to a fabrication yield of 70% (21/30, 95% Confidence Interval (CI) = 52.1–83.3%, Wilson score interval). The 21 successful nanopores had a mean diameter of 12.9 ± 12.8 nm (Number (n) = 21, Coefficient of Variation (CV) = 99.4%), with the largest pore measuring 38.8 nm. This overall yield demonstrates a robust and repeatable fabrication route for achieving sub-50 nm zero-depth nanopores, which are challenging to produce with conventional lithographic or etching-based methods.

The microscale failures primarily originated from poor electrical connections between the silicon chips and the board, which involved a combination of silver glue, aluminum tape, and single-core cable (Figure S12). This intermittent connectivity issue sometimes prevented the detection of the small currents that occur when the silicon blades are in tangential contact.

Figure 18b illustrates the size distribution of the fabricated nanopores. The orange bars represent the number of nanopores within four designated size ranges: 0–10 nm, 10–20 nm, 20–30 nm, and 30–40 nm, with 5 nm, 15 nm, 25 nm, and 35 nm denoting the respective bin centers. The distribution demonstrates that the majority of nanopores possess a diameter of approximately 5 nm. The prevalence of sub-10 nm pores, with nearly 35% in the 1–2 nm range, is particularly suitable for DNA sensing and potential sequencing applications. The blue curve depicts a fitted normal distribution, further confirming this trend.

The variation in nanopore size primarily stemmed from the electrical setup and our detection method for when the blades make contact. We have fabricated the blades using doped silicon wafers that do not have a specific resistivity. Our reference point for detecting the contact moment of the blades relies on a constant current of 100 pA. However, due to the varying contact resistance of the blades, the moment they stop can differ, sometimes causing the blades to cut through each other. This issue impacts the size of the pores.

To address this issue, we investigated measuring the tunneling current. We intended to measure the electrical current between the blades before they made contact. By doing so, after receiving the tunneling signal, we could adjust the blades with high precision until they touched each other [81]. However, this idea did not succeed. The reason for the failure is that a thin layer of SiO_2_ naturally forms on the surface of silicon in a room environment, which prevents the tunneling current from being detected [82].

### 3.4. Indentability of Silicon Micro-Blades

**Figure 19:**
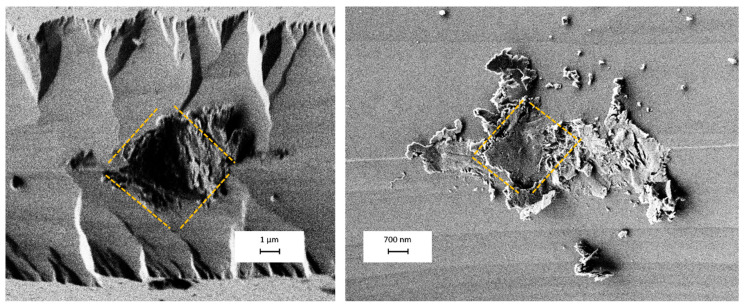
The SEM image shows silicon micro-blades after contact. These pictures are from two different experiments. The fraction of blades is easily visible, and they exhibit a 3D rhombic geometry, which is highlighted with an orange dashed line.

The contact points between the blades are very small, leading to significant stress at those locations. in addition, the silicon blades are inherently brittle and tend to break upon contact with one another. For the micropores, we can easily visualize the blades’ fracture effect. But what’s notable is that the blades do not break irregularly. When we examine the contact points, we can not only see the silicon particles but also observe rhombic-shaped indentations whose dimensions are compatible with the size of the pore (Figure 19).

However, in cases where we could fabricate nanopores, we did not observe any signs of fracture. The reason for this is that when the intrusion is around 20 nm or less, silicon exhibits elastic behavior. This means that for zero-depth nanopores with a size smaller than 100 nm, there is no concern about the blades breaking [83,84].

## 4. Conclusions

In this study, we investigated the fabrication of 3D rhombic zero-depth (RZD) solid-state nanopores using PDMS. To overcome challenges such as large pore size and low conductance reported in previous studies, we developed a novel approach involving PDMS molding at the intersection of two silicon micro-blades with nanoscale tip dimensions and wide vertex angles. We provided a detailed description of the micro-blade fabrication process, which utilized photolithography and a two-step KOH wet etching method. By utilizing conductive doped silicon wafers to fabricate the blades, along with a novel high-resolution nano-positioning system, a low-noise preamplifier-based electrical feedback loop, a temperature-controlled heat box with a PID regulator, active vibration isolation, shielding, low-noise electrical connectors, and appropriate silane treatments to reduce adhesion of PDMS to the silicon chips, we successfully fabricated sub-50 nm 3D RZD nanopores. Due to the challenges of using SEM for imaging and sizing of PDMS nanopores, we developed a finite element model and derived a mathematical correlation that enables us to size the RZD pores according to the electrical conductance and electrolyte concentration. The numerical model closely matched experimental results, with a mean absolute percentage error of 3.69%. Simulations further revealed that for channel lengths exceeding a critical value—which is proportional to the square root of the pore diameter—pore resistance becomes negligible. This allows for a linear relationship between electrical conductance and pore diameter. Remarkably, the zero-depth nanopores fabricated in this study exhibited conductance values 2.4 times greater than those fabricated by Ahmadi and Ardebili, demonstrating the effectiveness of our method. Given the ability to fabricate zero-depth nanopores using a low-cost mechanical process and PDMS—a biocompatible and flexible material—this work paves the way for the future of high-precision, tunable, and scalable solid-state nanopores.

## Data Availability

Data is contained within the article or Supplementary Material.

## Supplementary Materials

The following supporting information can be downloaded at: https://www.mdpi.com/article/10.3390/mi16121375/s1, Figure S1: SEM image of the silicon micro-blade; Figure S2: Schematic of the nano positioning setup; Figure S3: Schematic of the PDMS membrane; Figure S4: The rhombic geometry of the pore; Figure S5: Dimensions of the bending component; Figure S6: The contact of silicon micro-blades; Figure S7: The SEM image of a silicon blade coated with gold; Figure S8: Wearing off the gold layer; Figure S9: Schematic of the setup inside the heating (Faraday) cage; Figure S10: The SEM image shows a damaged silicon micro-blade; Figure S11: Using the active anti vibration table to minimize the mechanical noise; Figure S12: Schematic of the silicon chips and PMMA subplates arrangement; Figure S13: Nano-scale pores cannot be observed under an optical microscope; Figure S14: SEM image of nanopore; Figure S15: Geometry of the channels were used for simulation; Figure S16: The generated mesh; Figure S17: Variation of critical length with pore diameter; Table S1: Fabrication process flow of silicon micro-blades; Table S2: SEM parameters; Table S3: Failures; Table S4: Boundary conditions; Table S5: Constants and the parameters used in the simulation; Table S6: Mesh refinement study.

## Abbreviations

The following abbreviations are used in this manuscript:

RPS	Resistive Pulse Sensing
RZD	Rhombic Zero-Depth
DNA	Deoxyribonucleic Acid
PDMS	Polydimethylsiloxane
2D	Two-Dimensional
3D	Three-Dimensional
SEM	Scanning Electron Microscopy
TMCS	Trimethylchlorosilane
PFOTS	Trichloro-1H,1H,2H,2H-perfluorooctyl silane
MAPE	Mean Absolute Percentage Error
CI	Confidence Interval
CV	Coefficient of Variation
