# Supplementary material for: Fabrication of Sub-50 nm Three-Dimensional Rhombic Zero-Depth PDMS Nanopores with Enhanced Conductance via Silicon Micro-Blade Molding
Source: Micromachines (Basel). 2025 Dec 2;16(12):1375. doi: 10.3390/mi16121375 (PMC12735173; doi:10.3390/mi16121375)
Supplement: Supplementary file 1 [file micromachines-16-01375-s001.zip › micromachines-3951620-supplementary.pdf]

Supplementary material

Fabrication of Sub-50 nm Three-Dimensional Rhombic Zero-Depth PDMS Nanopores with Enhanced Conductance via Silicon Micro-blade Molding

Mohammad Matin Behzadi[1], Philippe Renaud[2] and Mojtaba Taghipoor[1*]

[1] Micro Nano Systems Laboratory (MNSL), Department of Mechanical Engineering, Sharif University of Technology, 1458889694 , Tehran, Iran., mohammadmatin.behzadi@mech.sharif.edu, Mtaghipoor@sharif.ir

[2] Microsystem Laboratory 4, École Polytechnique Fédérale de Lausanne (EPFL), 1015 Lausanne, Switzerland., philippe.renaud@epfl.ch

* Correspondence: Mtaghipoor@sharif.ir

1. Experiment

1.1. Process Flow

You can find the detailed description of process flow here:

Table S 1. Fabrication process flow of silicon micro-blades

Step	Process description	Cross-section after process
01	100/P/SS/01-05<br>Diameter: 100 mm<br>Thickness: 525 µm<br>Orientation: <100><br>Dopant: P – Boron<br>Resistivity: 0.1-0.5 ohm.cm<br>2 µm Wet ox	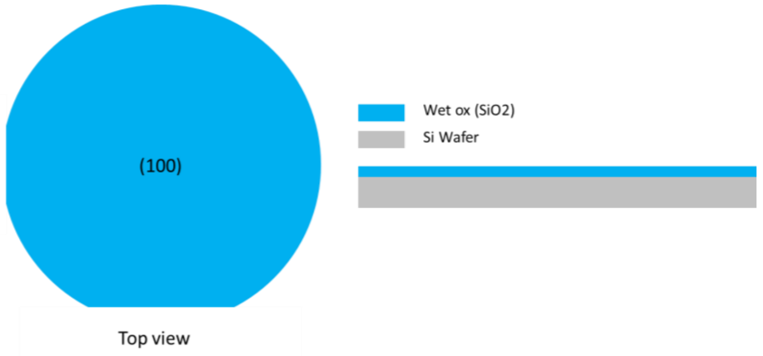
02	*HMDS Process*<br><br>150 C – 3 min	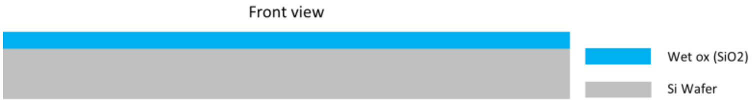
03	*Coating of Photo Resist*<br><br>PR: Az 1512, 2 µm	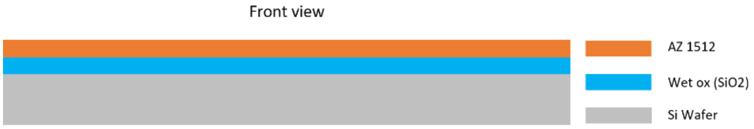
04	*UV Exposure with laser writer*<br><br>UV 405 nm<br>Invert Mode	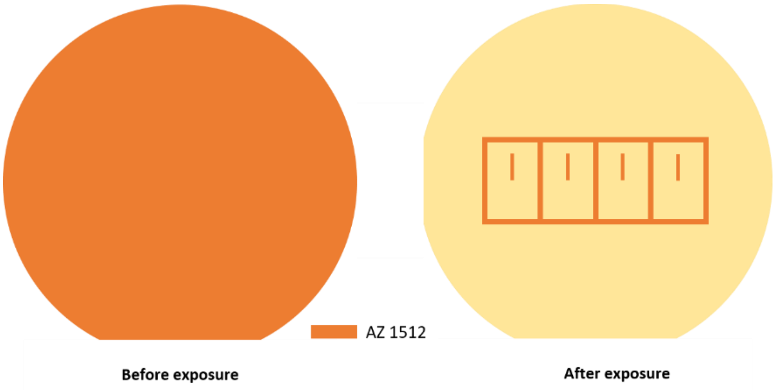
05	*Developement*<br><br>PR: Az 1512, 2 µm	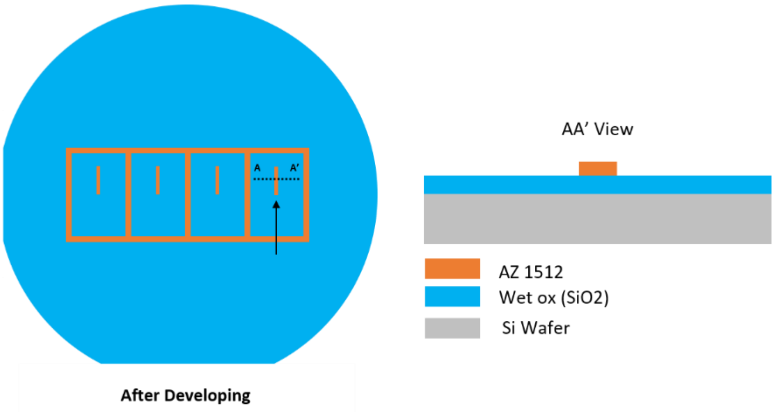

06	*Dry etching of SiO2*<br><br>Dry etching with C_4F_8<br>6 min	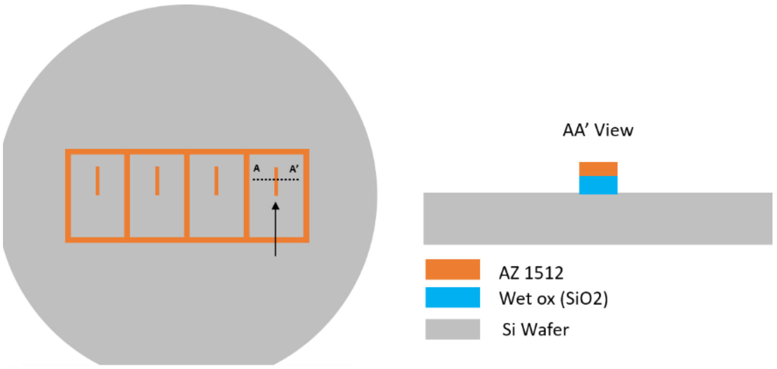<br>**After Removing SiO2**
07	*Stripping of Photo Resist with O_2 plasma*<br><br>10 to 15 min	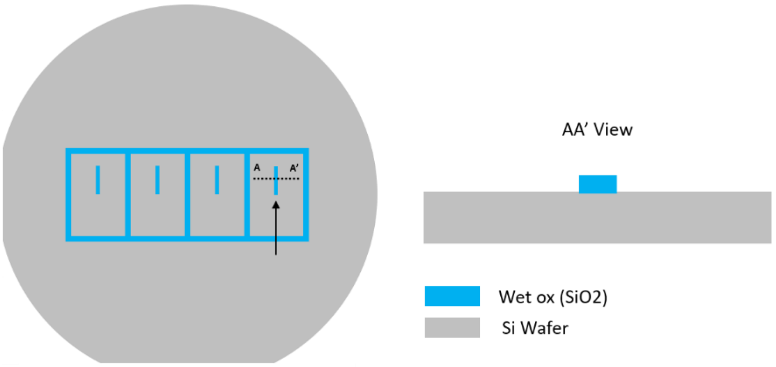<br>**After Removing PR**
08	*Wet Etching of Si*<br><br>KOH – 40% - 60 C<br>2 hours<br><br>Neutralization Process :<br>5 min rinsing in DI Water<br>+ 2 hours HCL 37 %<br>+ 5 min rinsing in DI Water	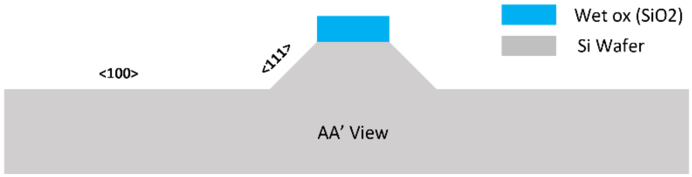
09	*Removing of SiO_2*<br><br>BHF<br>Room temp<br><br>40 min etching	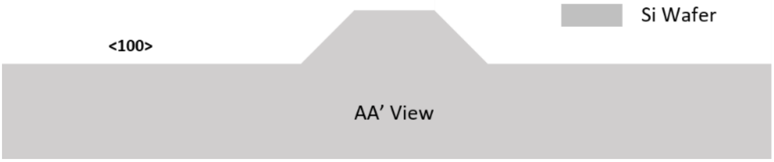

10	*Wet Etching of Si*<br><br>Etching of Si<br>KOH – 40% - 60 C<br>30 min<br><br>Neutralization Process	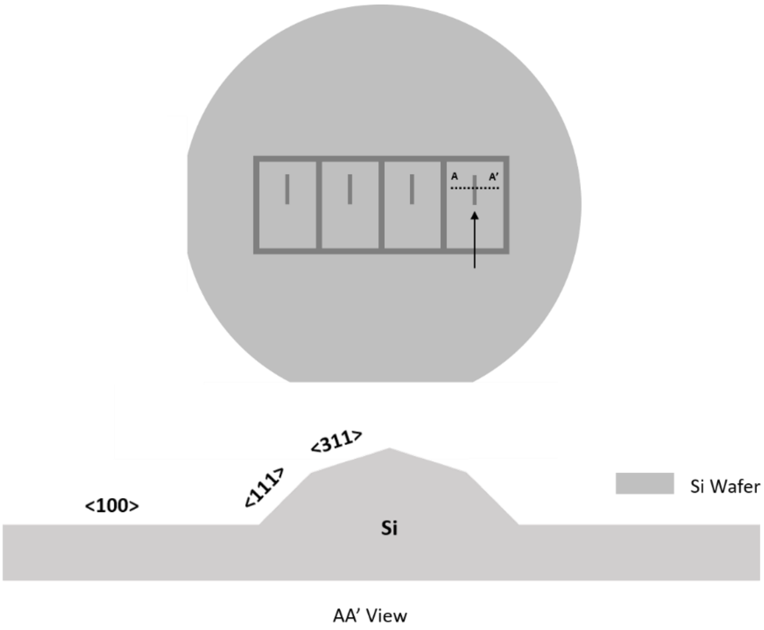

1.2. Geometry of Silicon micro blade

Fig S 1 illustrates the blade geometry, which has been examined using scanning electron microscopy (SEM). The blade is composed of <111> and <311> crystallographic planes The blades are 6 mm in length and approximately 40 µm in height

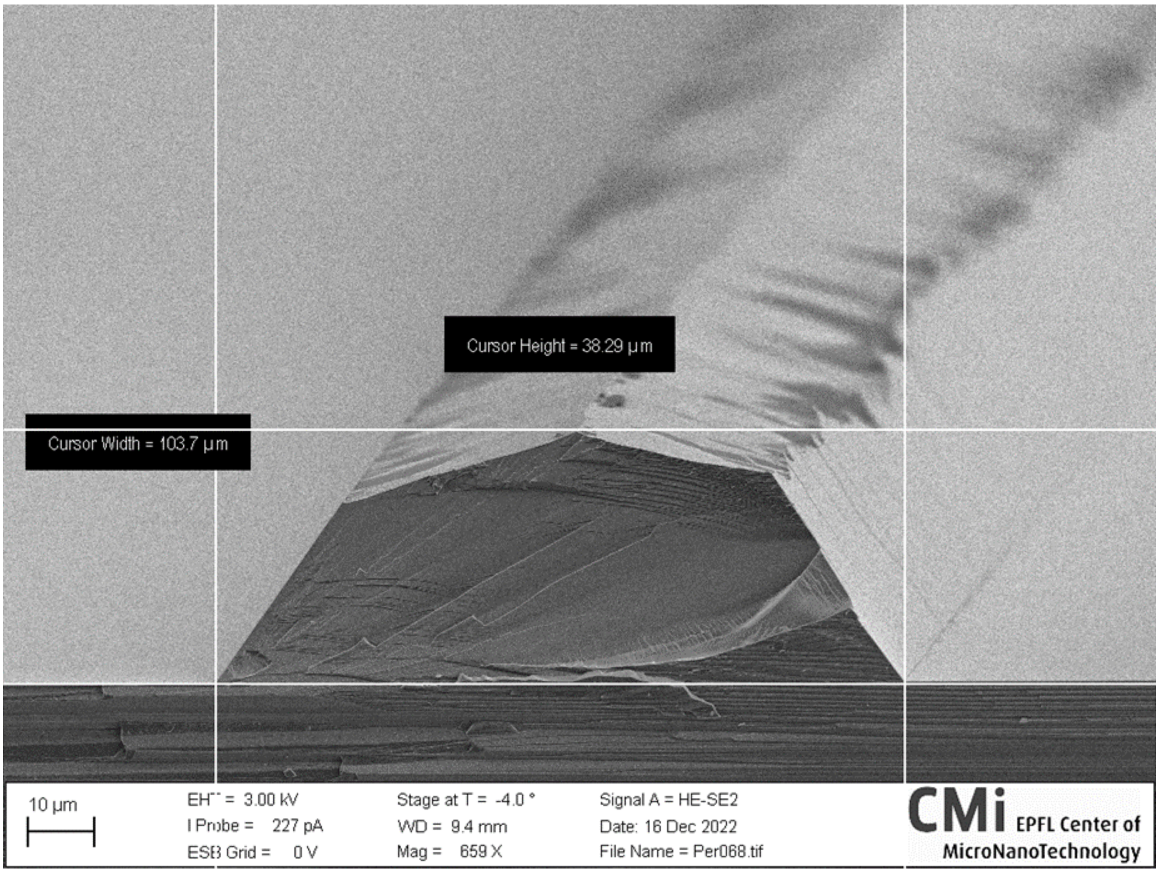

Fig S 1. A scanning electron microscopy (SEM) image of the silicon micro-blade was acquired without sputtering a conductive coating. The height and width of the micro-blade were directly measured from this image

1.3. Micro positioning setup function

The "Y" movement consists of two steps: coarse movement and fine movement:

- As mentioned in the manuscripts, the coarse movement is executed manually by turning the adjusting screw of the Y-Micro aligner (Fig S 2.a). This adjustment enables the holder of the motor to move up and down (Fig S 2.b).
- Once everything is set up and the blades are aligned with each other, we begin turning the adjusting screw (Fig S 2.c) until the blades are close enough together without making contact, which we can confirm visually (Fig S 2.d, e).
- This process typically takes just a few seconds, and we take care to avoid excessive coarse movement.
- For the fine movement, we utilize a piezo motor. This step can take around 20 to 30 minutes.

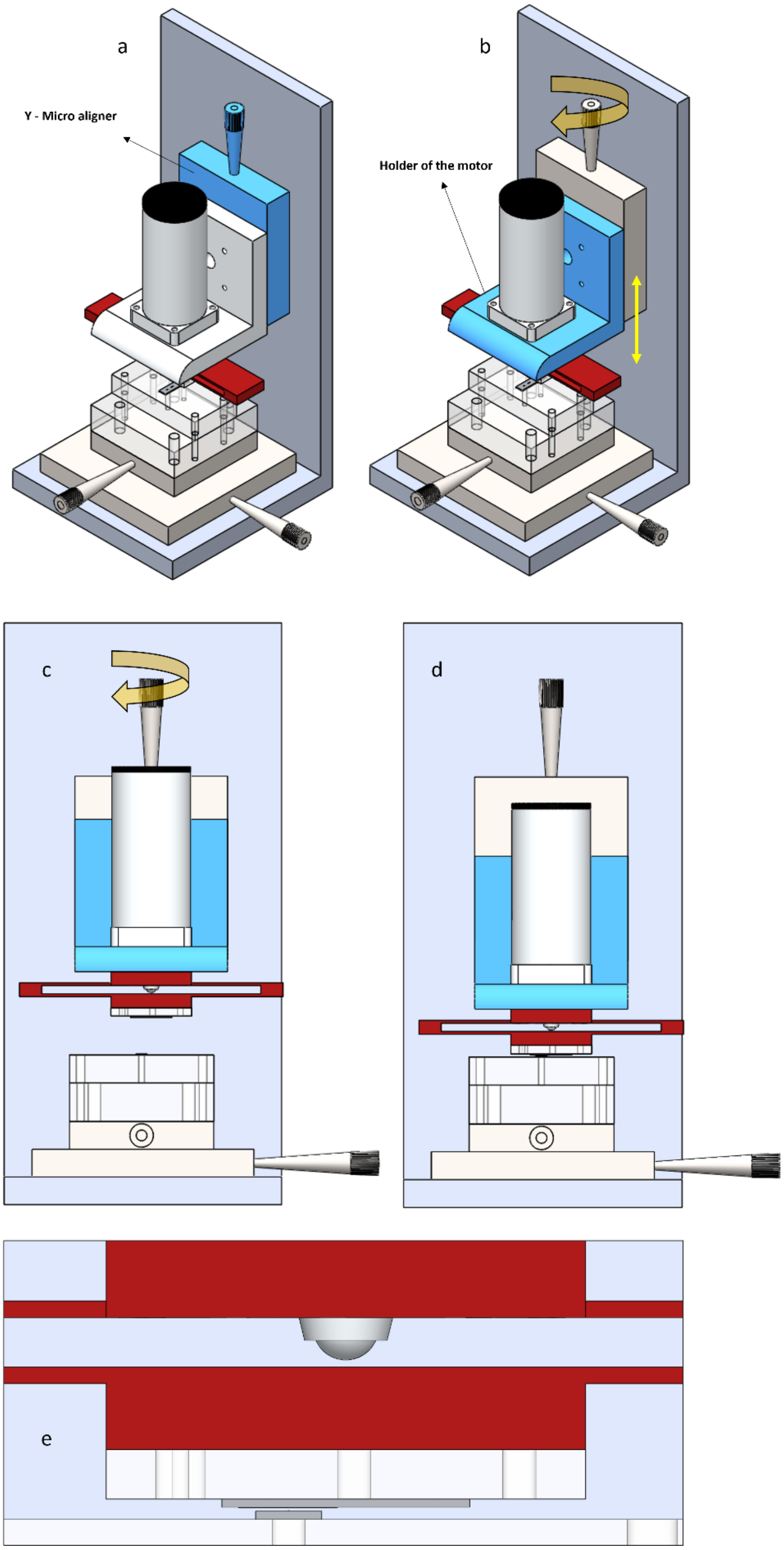

Fig S 2. Schematic of the nano positioning setup, illustrating both the manual coarse movement and subsequent fine alignment. (a) the coarse movement is executed manually by turning the adjusting screw of the Y-Micro aligner. (b) This adjustment enables the holder of the motor to move up and down. (c) Once everything is set up and the blades are aligned with each other, we begin turning the adjusting screw. (d) The coarse movement continues until the blades are in close proximity but not in contact. (e) Upon completion of the coarse movement, a narrow gap remains visible between the blades.

1.4. The PDMS membrane

The height of the blade is 40 µm, so when both blades are in contact, the gap between the chips measures 80 µm. As this gap is filled with PDMS, the nominal thickness of the membrane is 80 µm.

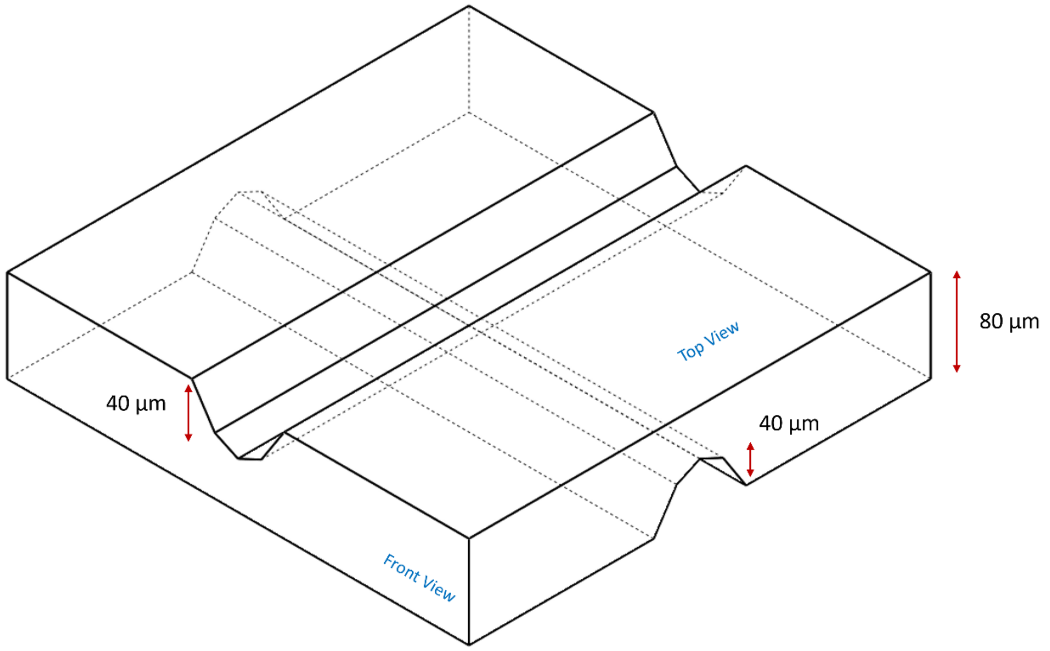

Fig S 3. Schematic of the PDMS membrane. The nominal thickness of the membrane is 80 µm.

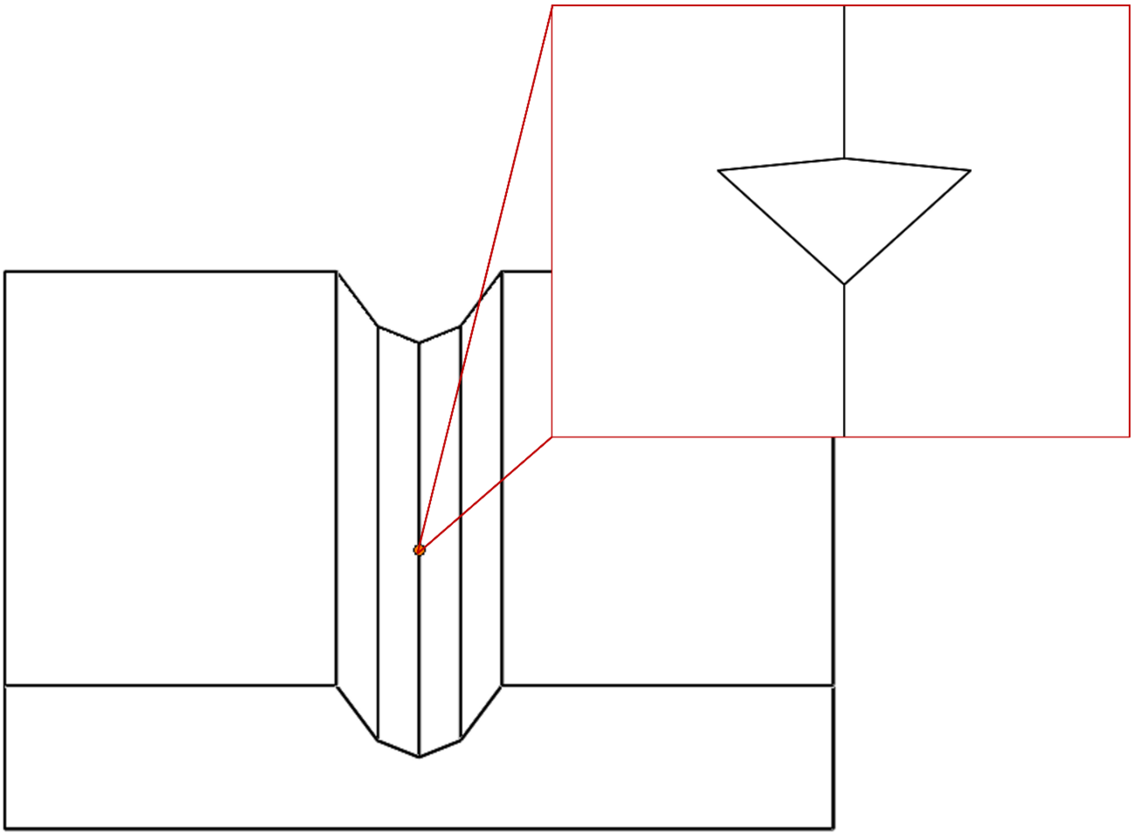

Fig S 4. The rhombic geometry of the pore

1.5. Bending component design

The dimensions of the bending component are illustrated in the figure below. This component is made of PLA and was produced using 3D printing.

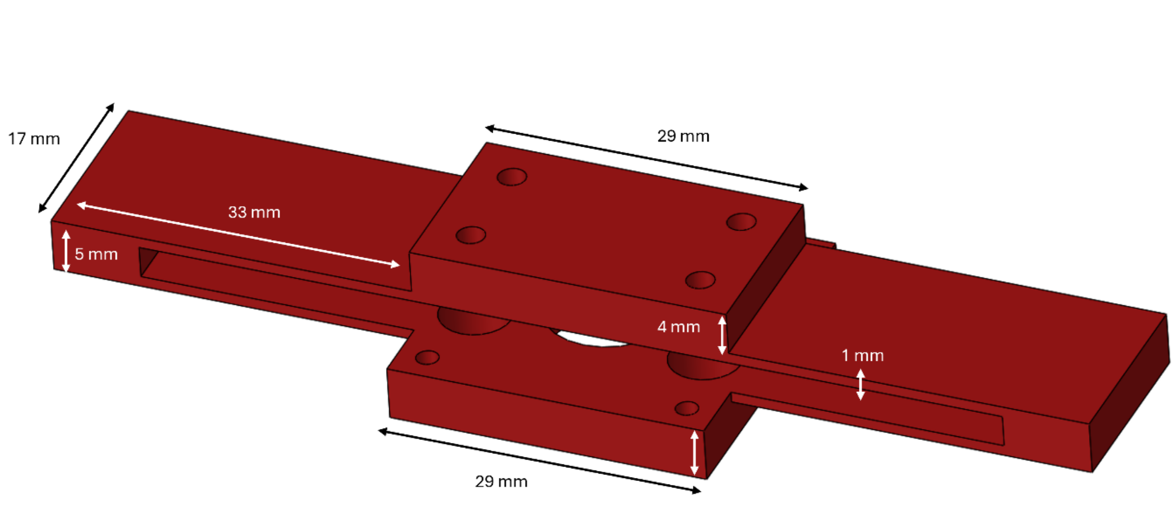

Fig S 5. Dimensions of the bending component

1.6. Blades collision

Upon blade contact, the control program issues a stop command to the piezo motor. The bending component enables this function by permitting vertical displacement of the upper chip along the Y-axis without inducing bending or tilting.

To verify that the chips are sufficiently parallel, we conducted a test without PDMS and took a photograph. In the zoomed image, it is clear that when the blades are in contact, the silicon chips remain parallel and well-aligned.

Additionally, it's important to note that if the horizontal plate bends, the silicon, being brittle, would likely break.

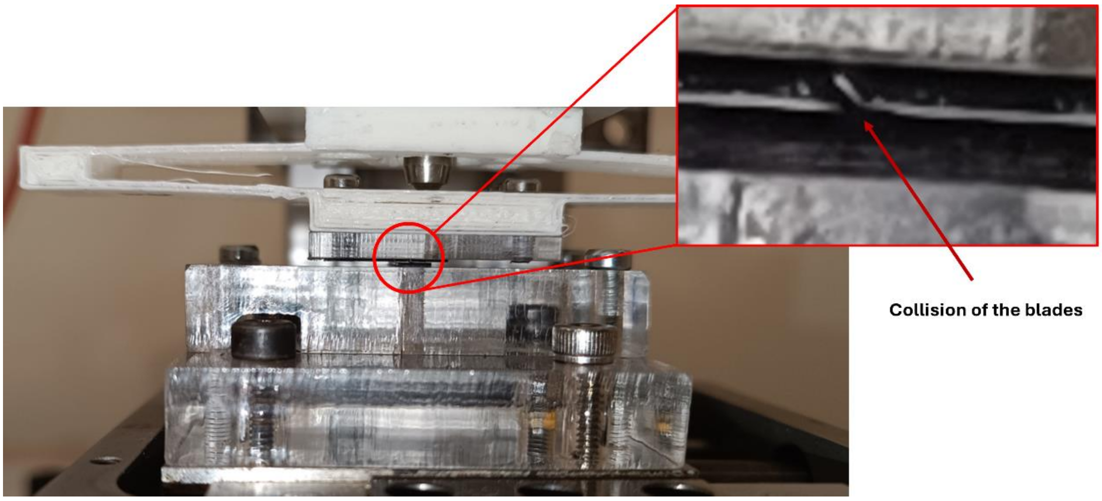

Fig S 6. The silicon micro-blades are in contact, and the chips are sufficiently parallel.

1.7. Why did we use doped Silicon wafers?

To enhance the conductivity of Si blades, that is the primary requirement for completing the electrical signal detection circuit, we initially applied a thin sputtered layer of chromium (5 nm) followed by a layer of gold (30 to 100 nm). The chromium layer promotes better adhesion for the gold layer; without it, the gold could be easily removed. The key advantage of using gold is that, while it is electrically conductive, PDMS does not adhere to gold after curing. This means there is no need for additional surface treatment to facilitate the detachment of the PDMS membrane.

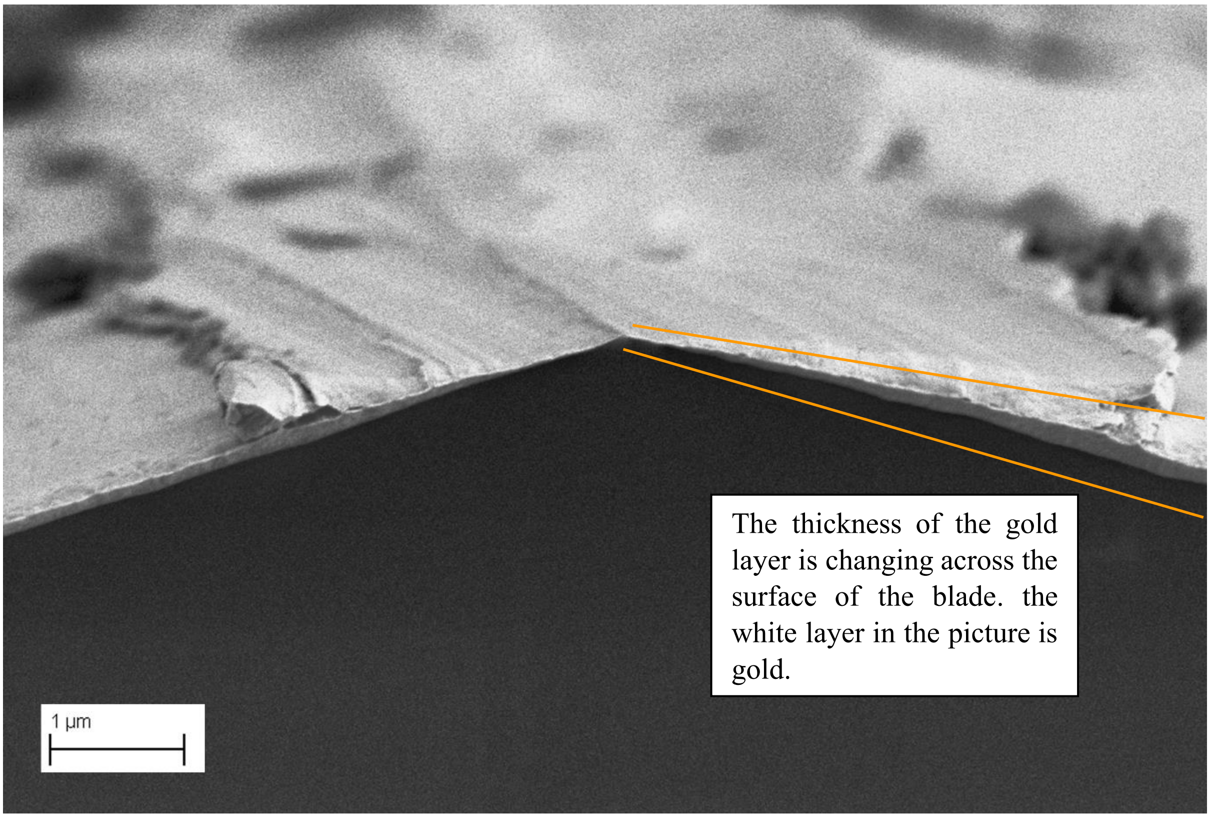

Fig S 7. The SEM image of a silicon blade coated with gold. Sputtering was employed to apply a thin layer of chromium (5 nm) and gold (30 to 100 nm) onto the blade. The thickness of the gold layer is not uniform, varies across the surface of the blade, and is minimum on the tip of the blade.

Our investigations revealed that the gold layer thickness on the blade is not uniform (Fig S 7). Furthermore, the high sharpness of the blades leads to the thin gold layer wearing off upon contact, which causes PDMS to adhere to the underlying layers. This adhesion can result in tearing and damage to the membrane during the separation of chips or the removal of the membrane (Fig S 8) and may lead to the formation of large pores. Consequently, we decided to use doped silicon wafers for the blades.

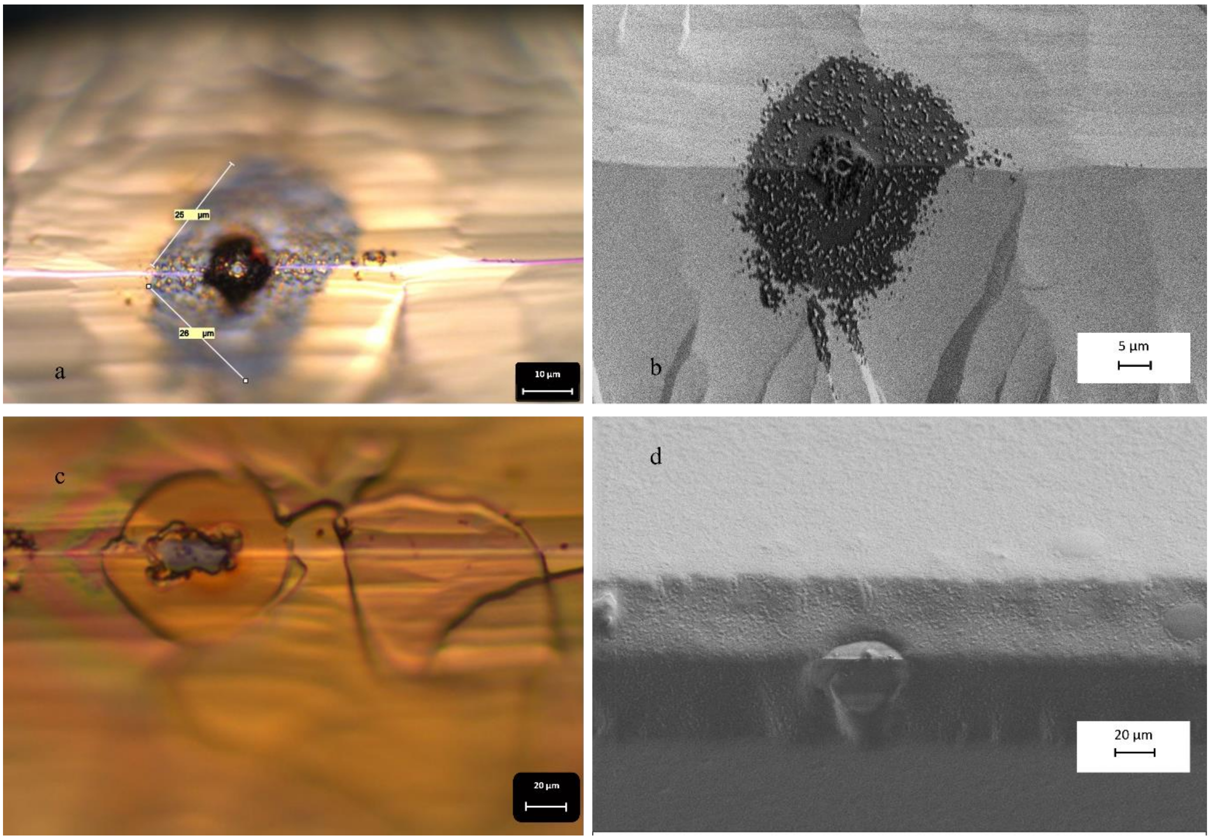

Fig S 8. Wearing off the gold layer. The results for two different tests are presented: (a), (c) Images a and c, taken with an optical microscope, clearly show that the gold layer has been removed. It should be noted that PDMS adheres to silicon or chrome; (b), (d) Images b and d are SEM pictures of blades a and c, respectively.

1.8. Heat Box

we utilize a thermostat equipped with a PID controller manufactured by CENGAGE. Two 300W U-shaped heaters are connected in series to the thermostat through a single-phase SSR-25DA relay. The temperature is measured using a K-01 type thermocouple. The entire system is housed in an aluminum box, which is grounded and also serves as a Faraday cage. With this setup, we can control and limit temperature variation to a maximum of 0.1°C. We set the temperature to 35°C and keep the system inside the box for 12 hours (Fig S 9).

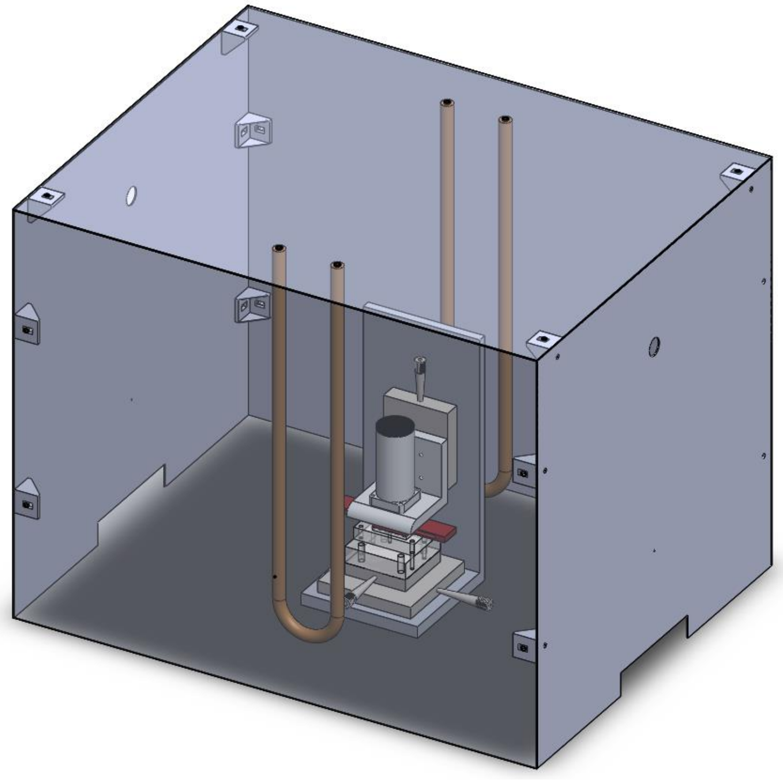

Fig S 9. Schematic of the setup inside the heating (Faraday) cage. Two U-shaped heating elements, rated at 220 V and 300 kW, are connected in series to generate heat within the aluminum box. A K-01 thermocouple is used to measure the temperature, while the thermostat controls the heater, turning it on and off according to the temperature readings. The temperature variation is maintained at 0.1℃, with a set point of 35℃. It takes approximately 12 hours to cure the PDMS.

1.9. Vibration

Vibrations can cause the blades to touch each other before they really come together, resulting in false stop signals. Additionally, if the blades do come into contact, vibrations may cause them to slide against one another, potentially damaging them and preventing the proper formation of the nanopore (Fig S 10).

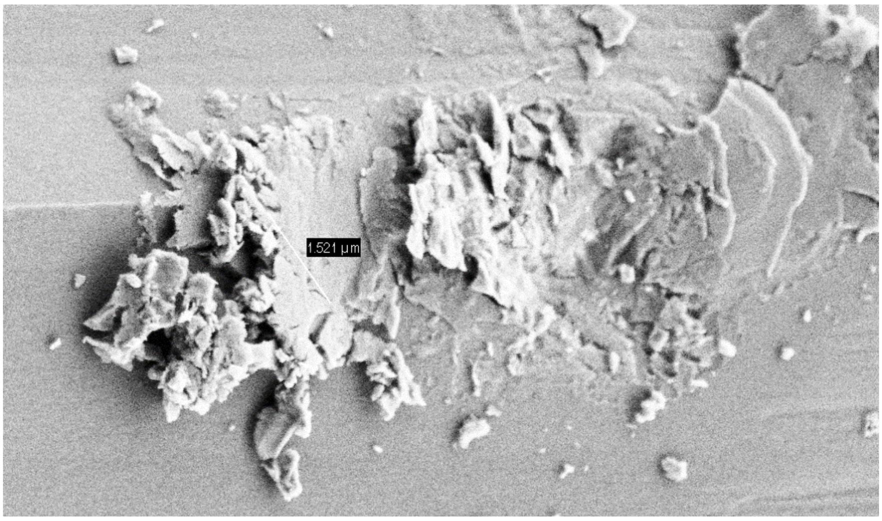

Fig S 10. The SEM image shows a damaged micro-Si blade. Due to vibrations, the blades have been sliding against each other, resulting in damage. If the blade loses its sharpness, it can no longer be used to create nanopores.

Initially, we conducted our experiments on the 5th floor using a regular table. To dampen some of the vibrations, we attempted to use a heavy stone plate, but this was not successful.

To address this issue, we relocated our setup to another lab on the basement level (-1) and placed it on an active anti-vibration table (Fig S 11. Using the active anti vibration table to minimize the mechanical noise. To avoid any unpredictable vibrations, we decided not to put the laptop on the table, as we believed its fan could produce vibrations.

To minimize any other potential sources of mechanical noise, we carried out our experiments at night when the lab and the building were less crowded. Following these protocols helped us eliminate the previously observed damage.

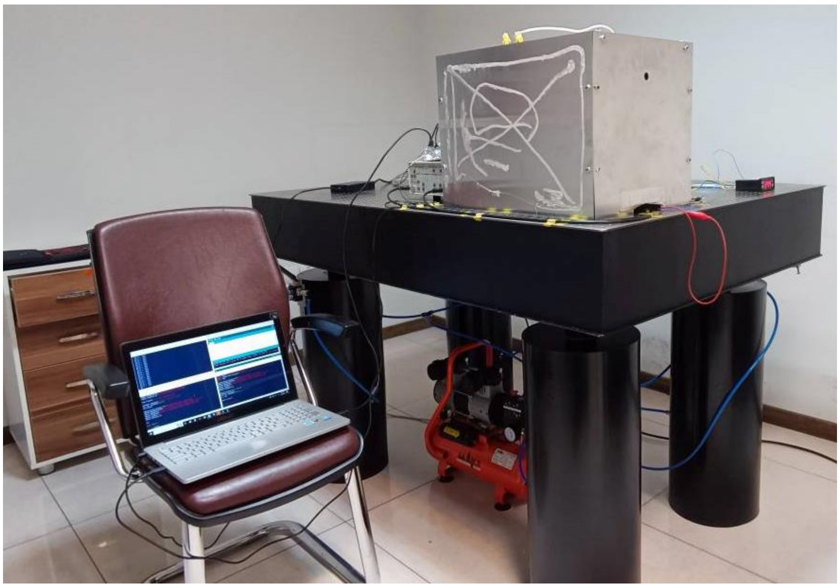

Fig S 11. Using the active anti vibration table to minimize the mechanical noise

1.10. Separating the chips

The "Upper" and "Lower chips" are connected to the PMMA subplates (Fig S 12). In addition to the chip surfaces, PDMS is spread over certain areas of the PMMA plates. After the PDMS has cured and the test is complete, separating the chips results in tearing of the PDMS membrane, as both the chips and PMMA plates have the same level of adhesion to the PDMS.

To address this issue, we can coat the upper subplate with sodium stearate. This treatment reduces the adhesion of PDMS to PMMA. As a result, when we remove the chips, the membrane adheres more effectively to the lower subplate. This helps it detach from the upper chip and the upper subplate while remaining on the lower chip.

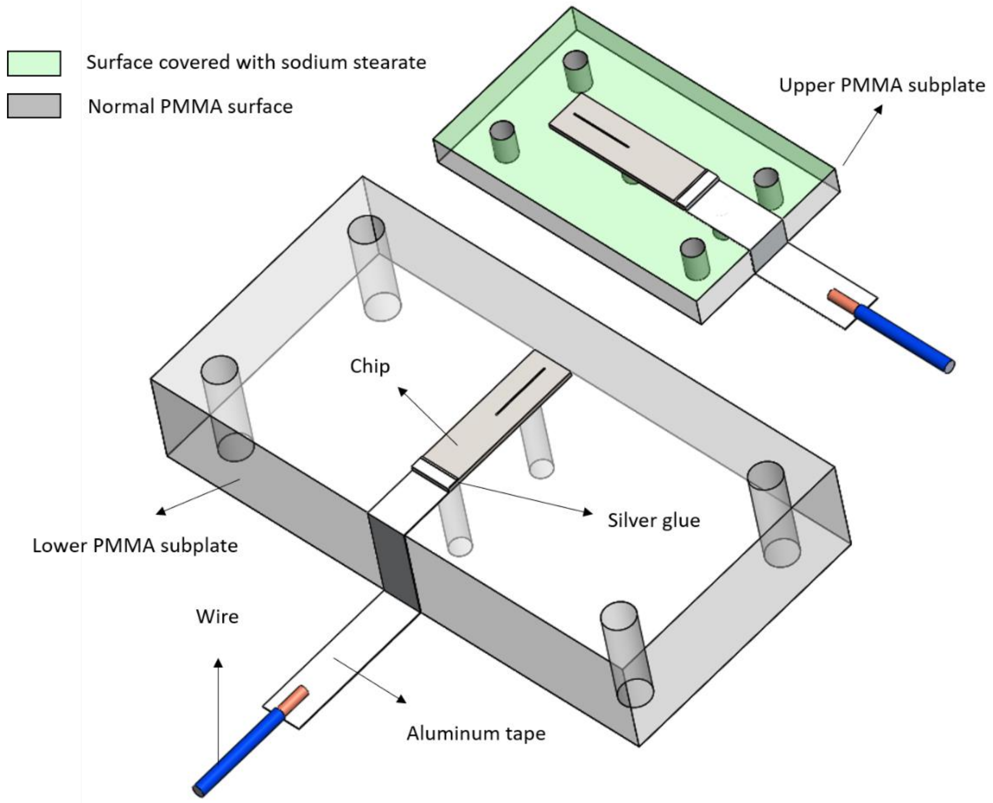

Fig S 12. Schematic of the silicon chips and PMMA subplates arrangement. Both the upper and lower subplates are made of PMMA, while the chips are composed of silicon and coated with TMCS. The key to maintaining the cured PDMS membrane on one of the chips, while allowing the separation of the chips, lies in the differing adhesion levels. The upper subplate, shown in green, is treated with sodium stearate. Because the bare PMMA surface exhibits stronger adhesion to PDMS, it helps keep the membrane attached to the lower chip and lower subplate. Wire and aluminum tape serve as connectors. To enhance the electrical connection between the aluminum tape and the silicon chip, conductive silver glue is utilized.

1.11. Visualizing Nano pores using optical microscope

By mitigating error factors, we successfully fabricated nanopores. As naanopores are not visible with optical microscopes (Fig S 13), scanning electron microscopy is necessary for their visualization.

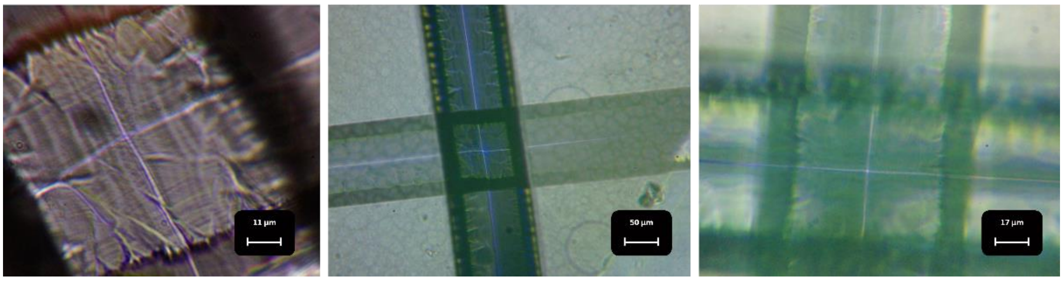

Fig S 13. Nano-scale pores cannot be observed under an optical microscope. The theoretical minimum feature size that can be resolved using an optical microscope is approximately 200 nm. However, actual resolution can be impacted by factors such as lens quality, light intensity, and image processing techniques. For measuring 3D rhombic zero-depth nanopores, scanning electron microscopy (SEM) or indirect sizing methods should be utilized.

1.12. SEM setting

Because PDMS is electrically insulating, it is necessary to sputter a thin layer of gold onto the membrane before SEM imaging. Also, when operating the SEM, it is im-portant to use low-voltage mode; otherwise, the electron charge could damage the membrane and cause variations in pore size. Fig S 14 illustrates a nanopore with an approximate size of 300 nm.

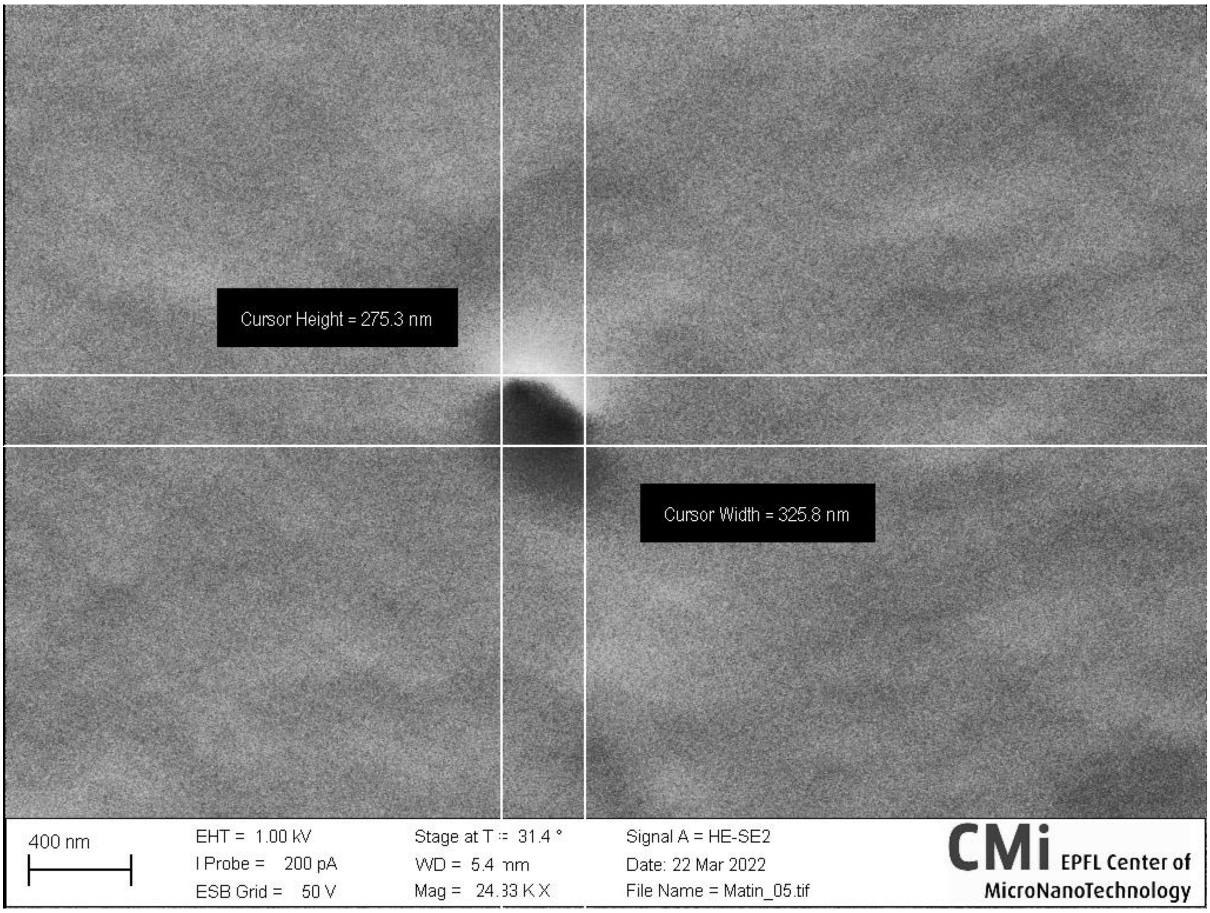

Fig S 14. The SEM image shows a rhombic zero-depth nanopore in a PDMS membrane, with a pore size of approximately 300 nm. To enhance image quality, a 5 nm layer of gold was sputtered onto the membrane.

The other SEM settings used were as Table S 2:

Table S 2. SEM parameters

Parameter	Value
Accelerating Voltage (EHT)	1.00 kV
Beam Current (I Probe)	200 pA
Signal Detector	HE-SE2
Magnification	24.33 KX
Working Distance (WD)	5.4 mm
Stage angle	31.4 °C
ESB Grid Voltage	50 V

1.13. Failures

A summary of unsuccessful fabrication attempts is provided in Table S 3, with the cause of failure for each sample detailed in the description column.

Table S 3. Failures

Sample	description
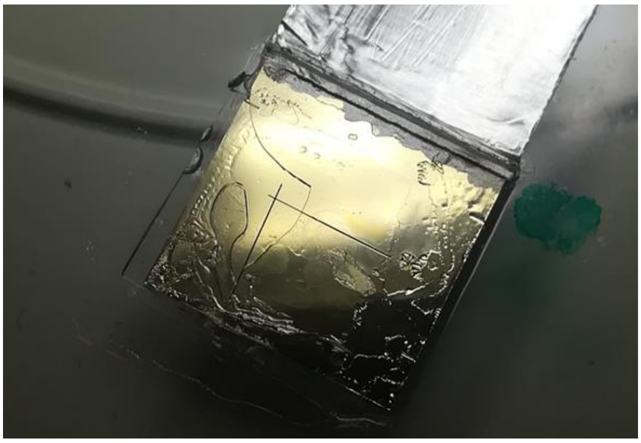	Membrane tearing occurred due to similar adhesion strength between the PDMS membrane and both the upper and lower chips. This problem and its solution are detailed in Section 1.9 of the Supplementary Materials.<br><br>* In our initial tests, a different silicon chip geometry was used, which had been sputtered with 5 nm of chromium and 30 nm of gold.
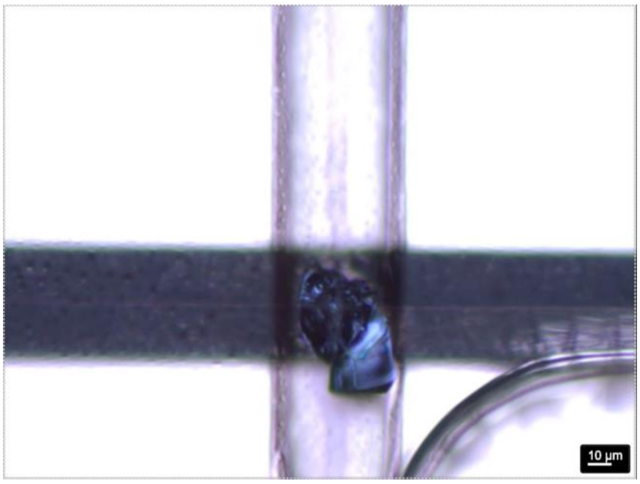	Membrane tearing occurred due to the adhesion of the PDMS to the silicon blade. This instance exemplifies a rare challenge that can occasionally arise when using TMCS for silicon surface treatment.<br><br>PFOTS and TMCS are compared in the section 2.5 of the manuscript.
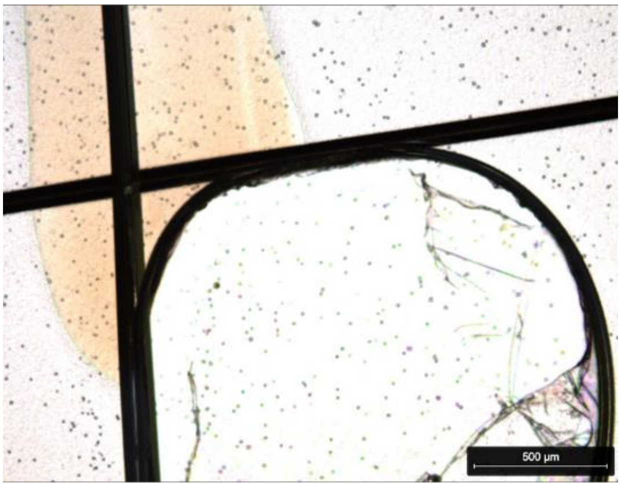	Bubble formation resulted from insufficient degassing of the PDMS. The PDMS must be degassed twice: first after mixing with its curing agent, and again after being poured onto the chip.

<table>
<tr>
<td>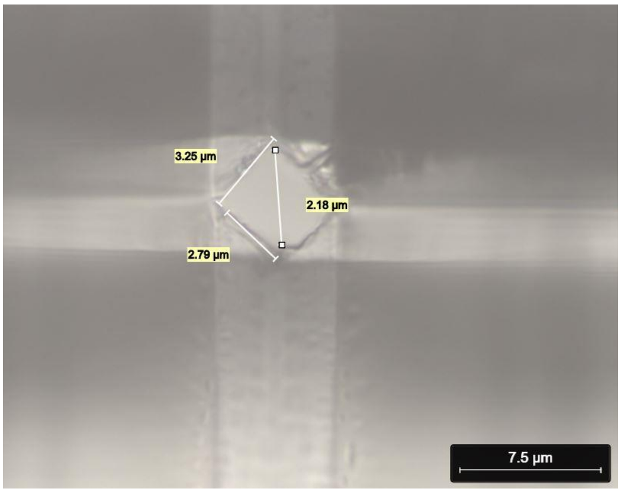
</td>
<td>Micro scale pores are formed because of issues such as vibration, temperature variation, electrical noise or choosing of large stop current.</td>
</tr>
<tr>
<td>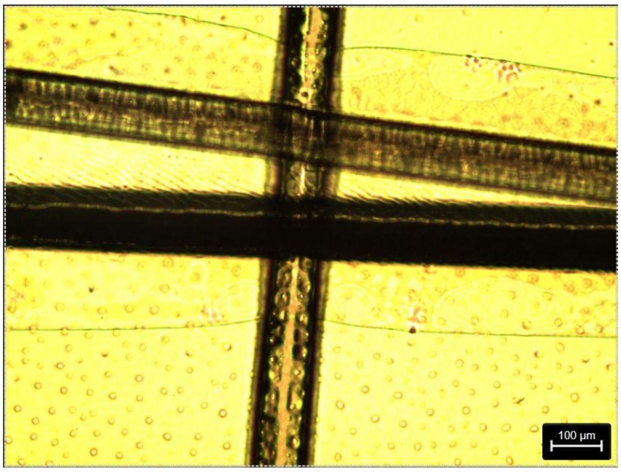
</td>
<td>One of the chips moved during the PDMS curing process due to external vibration. This misalignment resulted in the upper channel being formed twice.</td>
</tr>
<tr>
<td>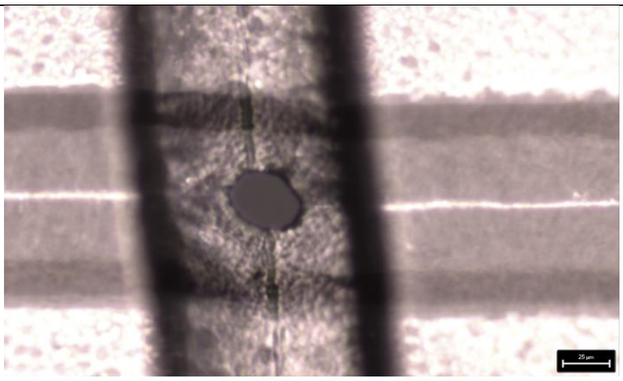</td>
<td>When the blades collide, small PFOTS droplets near the contact point merge to form a larger droplet. This aggregation of PFOTS droplets prevents PDMS from filling the area, leading to the creation of large circular pores.
This issue and it’s solution is explained in detail in the section 2.5 of the manuscript.</td>
</tr>
</table>

2. Simulation

2.1. Geometrical Parameters

The schematic diagram of simulated 3-dimensional geometry is demonstrated in Fig S 15. The Reynolds number is always less than 1 (Re < 1), implying laminar flow. Accordingly, the flow field can be assumed to be symmetrical horizontally and vertically, thus a quarter of the entire flow domain is used in this simulation, as shown in Fig S 15 (a). Boundary conditions are available in Table S 4. Constant values and parameters used in this simulation are available in Table S 5.

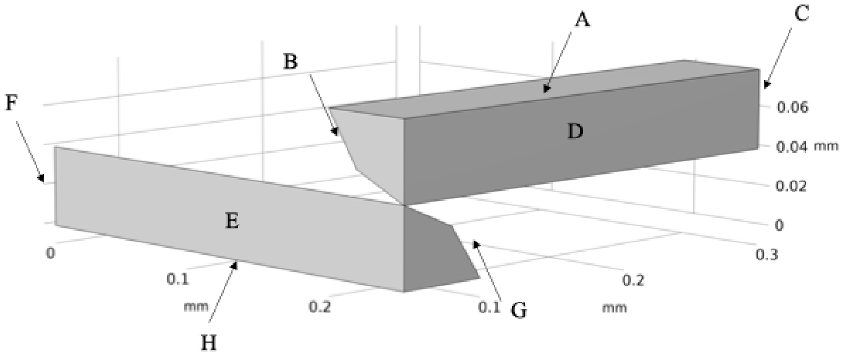

a

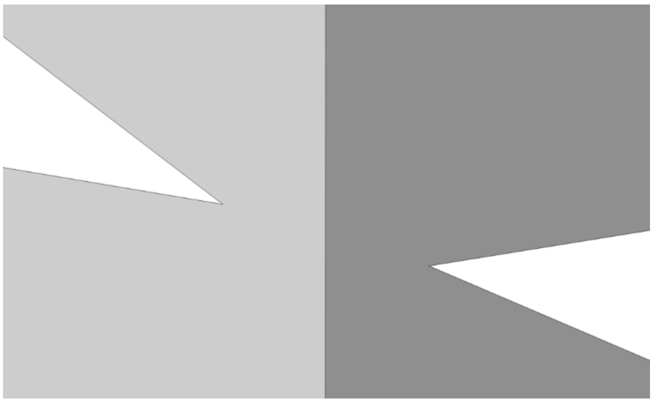

b

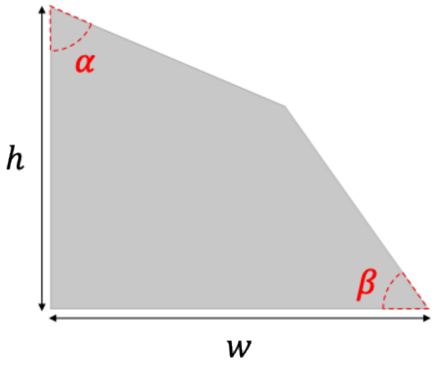

c

Fig S 15. Geometry of the channels were used for simulation: (a) The simulated geometry; (b) The detailed view of the nanopore; (c) geometrical parameters of blades.

Table S 4. Boundary conditions for Poisson, Nernst-Planck and Stokes Equation.

Boundary	Poisson Equation	Nernst-Planck Equation	Stokes Equation
A	$V = 0.25\ V$	$c_{0,cp} = c_{K^+}$<br>$c_{0,cn} = c_{Cl^-}$	$p_0 = 0\ Pa$
B, C, F, G	$n.(\epsilon_0\epsilon_r E) = \rho_{s,w}$	$-n.(J_j + uc_j) = 0$	$u = 0$
D, E (Symmetry)	$n.E = 0$	$-n.(J_j + uc_j) = 0$	$u.n = 0$<br>$K_n.(K_n.n)n = 0$<br>$K_n = Kn$
H	$V = 0\ V$	$c_{0,cp} = c_{K^+}$<br>$c_{0,cn} = c_{Cl^-}$	$p_0 = 0\ Pa$

Table S 5. Constants and the parameters used in the simulation.

Parameter	Value	Unit	Description
D	100 - 500	nm	Pore diameter
l	0.5	mm	Blade length
h	0.04	mm	Blade height
w	0.05	mm	Maximum blade width
α	67	degree	Apex angle of the blade
β	54.7	degree	Lower interior angle of the blade
T	298	K	Temperature
$\rho_{s,w}$	0	C/m^2	Nanopore surface charge density
D_{K^+}	1.957×10^{-9}	m^2/s	Diffusion coefficient of K^+
D_{Cl^-}	2.032×10^{-9}	m^2/s	Diffusion coefficient of Cl^-
c_{K^+}	250	mol/m^3	Concentration of K^+
c_{Cl^-}	250	mol/m^3	Concentration of Cl^-
z_{K^+}	+1	1	The charge of K^+
z_{Cl^-}	-1	1	The charge of Cl^-
ϵ_r	80	1	Relative permittivity
ϵ_0	8.8542×10^{-12}	F/m^2	Vacuum permittivity
η	1.0016	mPa.s	Dynamic viscosity of water
F	96485.33	C/mol	Faraday constant
e	1.602×10^{-19}	C	Electron charge
R	8.3145	J/(mol.K)	Gas constant

2.2. Governing Equations

In terms of mathematical modelling, a set of coupled equations, including Poisson, Nernst-Planck, and Stokes, was employed (Eq. (S1)-(S3)). Additionally, the fluid flow was considered incompressible in continuity equation (Eq. (S4)). The main governing equations are formulated as below:

$$\nabla.(\epsilon_0\epsilon_r E) = F\sum_i z_i c_i \tag{S1}$$

$$\nabla.(-D_i\nabla c_i - z_i u_{m,i} F c_i \nabla V) + u.\nabla c_i = 0 \tag{S2}$$

$$\nabla.(-pI + \eta(\nabla u + (\nabla u)^T)) + \left(F\sum_i z_i c_i\right)E = 0 \tag{S3}$$

$$\nabla.(u) = 0 \tag{S4}$$

where $\boldsymbol{\epsilon_0}$ is the vacuum permittivity, $\boldsymbol{\epsilon_r}$ is the relative permittivity, $\boldsymbol{E}$ is the electric field, F is the Faraday constant, $\boldsymbol{z_i}$ is the valence of ith ionic species, $\boldsymbol{c_i}$ is the concentration of ith ionic species, $\boldsymbol{D_j}$ is the diffusion coefficient of the electrolyte solution, $\boldsymbol{V}$ is the electric potential, $\boldsymbol{u}$ is the electrolyte solution velocity, $\boldsymbol{p}$ is pressure of the electrolyte solution, $\boldsymbol{I}$ is the identity matrix, and $\boldsymbol{\eta}$ is the viscosity of the electrolyte solution. $\boldsymbol{u_{m,j}}$ is the mobility, which is computed from Nernst-Einstein relation, as illustrated in Eq. (S5):

$$u_{m,i} = \frac{D_i}{RT} \tag{S5}$$

where $\boldsymbol{R}$ is the gas constant, and $\boldsymbol{T}$ is the temperature of the electrolyte solution. Considering A as an arbitrary cross-sectional area, ionic current through the nanopore can be calculated according to Eq. (S6):

$$I_P = \iint_A F\sum_i z_i\left(-D_i\nabla c_i - z_i u_{m,i} F c_i \nabla V + u.\nabla c_i\right) dA \tag{S6}$$

2.3. Mesh Independence Study

For the mesh independence study, we have assumed one coefficient, α, to refine the mesh in the nanopore domain. α indicate the coefficient divided by the maximum and minimum size of the elements in the nanopore domain, respectively. The maximum element growth rate in the nanopore domain is 1.3.

For blades domain, the maximum and minimum element sizes are chosen 5 µm and 50 nm, respectively. Then, we have increased α values to have finer mesh in the nanopore domain. The error is computed concerning the most-refined mesh, and the optimized value is adopted. The selected values for α are available in Table S 6. The generated mesh for the geometry is available in Fig S 16, consisting of 195,363 free tetrahedral elements. As shown in Fig S 16.b , the nanopore domain has finer elements. The maximum and minimum element sizes are 10 nm and 0.1 nm, respectively.

Table S 6. Mesh refinement study for α (c_0 = 250 mol/m3, D = 100 nm)

α	No. of elements	I (µA)	Error (%)
100	106,729	0.09599893	0.09941771
200	118,887	0.09590358	0.07169412
300	144,386	0.09583487	0.04383831
400	167,134	0.09579288	0.0067327
500	195,363	0.09578643	-0.0094174
600	219,928	0.09579545	0.02845658
700	277,772	0.0957682	0.00729514
800	345,129	0.09576121	0.01016397
1000	482,967	0.09575148	0

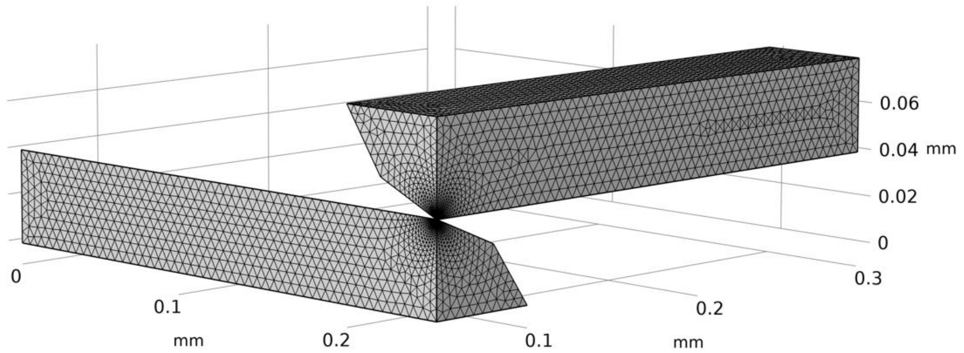

a

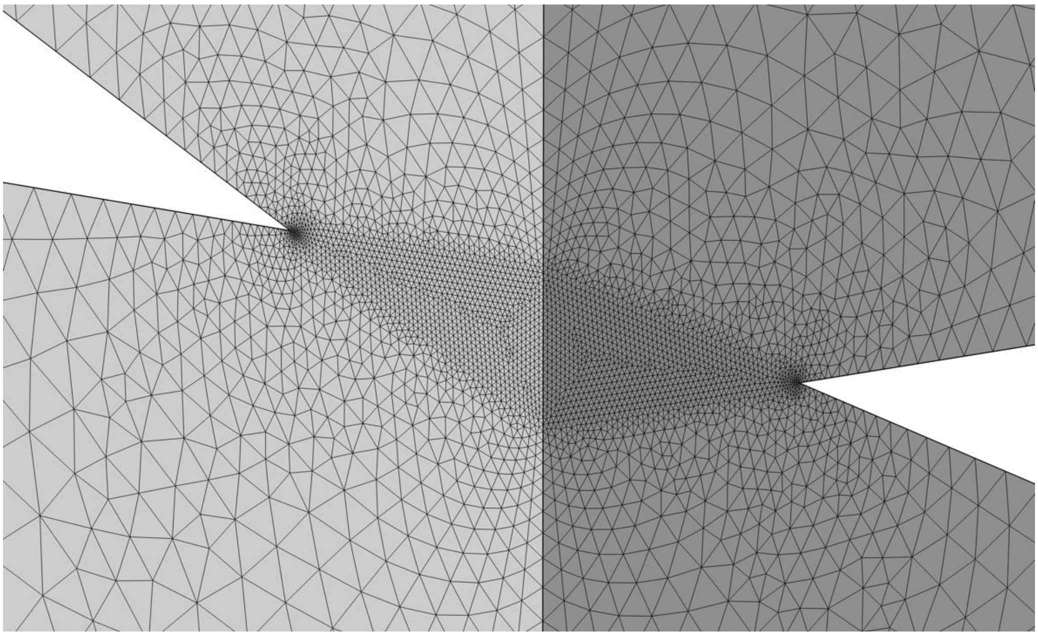

b

Fig S 16. The generated mesh: (a) the whole domain; (b) the nanopore domain.

2.4. Critical length

Figure 18 illustrates that reducing the channel length below a certain value, termed the "Critical length," results in a decrease in pore conductance. In other words, pore resistance becomes negligible only when the channel length exceeds the Critical length.

The derived Equation (2) estimates the Critical length (L_C) for the specific geometry being studied, indicating that the critical length is proportional to the square root of the pore diameter (D).

The Critical length (LC) is defined as the channel length beyond which the change in pore conductance is less than 1%. When the channel length is smaller than the critical value, the G-D relation is nonlinear.

The only variable in this analysis is the pore diameter (D). For various values of D, we determined the critical length, fitted a curve to these values, and derived the governing equation.

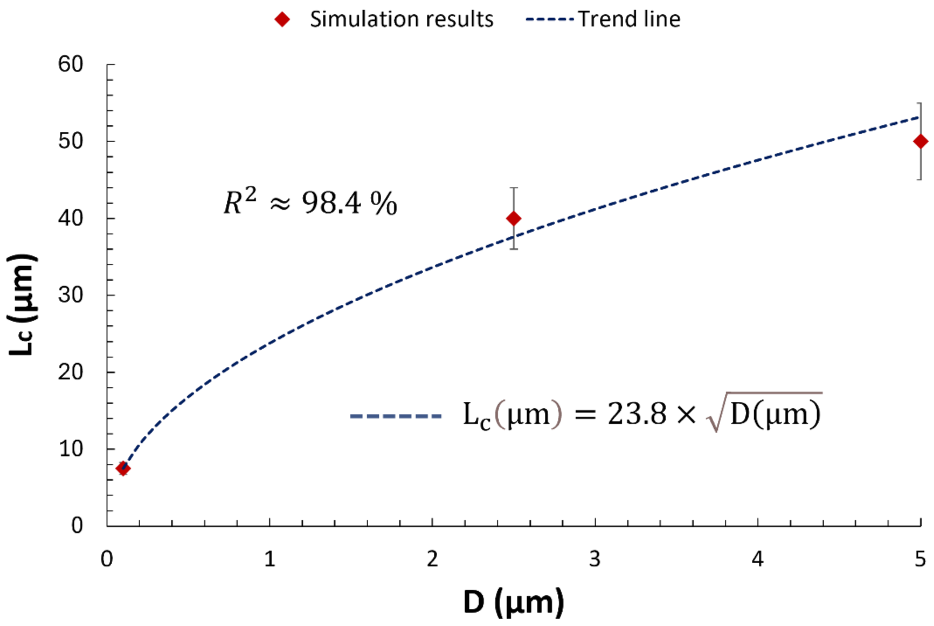

Fig S 17. Variation of critical length with pore diameter.
